# NeoThy™ immune-humanized mice can produce anti-drug antibodies to support immunogenicity assessment for biological drug products

**DOI:** 10.3389/fimmu.2026.1851898

**Published:** 2026-08-12

**Authors:** Sepideh Gharaie, Robyn E. Becker, Elizaveta A. Svyatova, Megan K. Chow, Nicholas A. Smith, Adam J. Bell, Mohit Pratap Singh, Kathleen L. Gabrielson, Katherine I. Shea, Janell Richardson, Kristina E. Howard

**Affiliations:** 1 Division of Applied Regulatory Sciences, Office of Clinical Pharmacology, Office of Translational Sciences, Center for Drug Evaluation and Research, U.S. Food and Drug Administration, Silver Spring, MD, United States; 2 Department of Molecular Analysis, Taconic Biosciences, Rensselaer, NY, United States; 3 Department of Molecular and Comparative Pathobiology, School of Medicine, Johns Hopkins University, Baltimore, MD, United States; 4 Department of Research & Development, Taconic Biosciences, Rensselaer, NY, United States

**Keywords:** anti-drug antibody, IFNβ, immune-humanized mice, immunogenicity, infliximab

## Abstract

**Introduction:**

Assessment of immunogenicity for new therapeutic protein products and biosimilars is an important aspect of drug development and the safety evaluation process. Conventional animal models typically do not reliably predict immunogenicity, as the human drug is seen as foreign in the animal model. To address this need, we conducted a proof-of-concept study with neonatal thymus (NeoThy™) immune-humanized mice using drug products known to have higher rates of immunogenicity in patients. NeoThy immune-humanized mice develop a fully engrafted human immune system that includes a human thymus for proper T cell development capable of recognizing human versus foreign proteins.

**Objective:**

This study used infliximab and interferon-beta (IFN-β) as monotherapies and in combination to determine if NeoThy immune-humanized mice could produce specific adaptive immune responses to the drugs.

**Methods:**

NeoThy immune humanized mice were generated at two different locations, creating six experimental groups. Mice received infliximab, IFN-β, a combination of infliximab and IFN-β, positive or negative control with treatment schedule appropriate to the therapeutic. Peripheral blood/serum was collected on day 0, 21, 42, and 63; tissues (bone marrow, spleen, lymph nodes) were harvested at necropsy (day 63). Immune reconstitution and treatment effects were assessed with flow cytometry, proliferation, total IgM/IgG isotyping, anti-drug antibodies (ADA) ELISAs for infliximab and IFN-β, and histopathology.

**Results:**

NeoThy immune-humanized mice showed broad and ample human immune reconstitution with donor-dependent differences noted. We observed immune activation and B cell class switching including IgG_1–4_ in most donors. In several cases, humanized mouse serum immunoglobulin levels approached or exceeded those from normal human serum, which was evaluated as a control. Binding ADAs to IFN-β and neutralizing ADAs to infliximab, were detected in multiple mice in a donor- and treatment-dependent manner.

**Conclusions:**

NeoThy immune-humanized mice mount human-like, donor-dependent immune responses to biologics, including class switching, antigen-specific proliferation, and ADA formation. These preliminary findings suggest that NeoThy immune-humanized mice have the biological ability to help assess immunogenicity risk, and other immunosafety considerations for biological drug products.

## 1 Introduction

Biological drug products, ranging from monoclonal antibodies to therapeutic peptides, are used to treat a wide variety of illnesses, including cancer, autoimmune diseases, and infectious diseases. These products have revolutionized therapy for many diseases; however, unlike small-molecule drug products, they can be recognized by the patient’s immune system. When the patient’s immune system responds to these therapeutic proteins with anti-drug antibodies (ADAs), it is called “immunogenicity”. Immunogenicity in this context means that the drug products could be neutralized by binding the epitope required for the drugs’ effect [neutralizing anti-drug antibodies (nADAs)], or they may simply bind the drug product [binding anti-drug antibodies (bADAs)] but still be responsible for alterations in drug clearance and/or causing hypersensitivity (1–4). The most common method for assessing whether patients have mounted this type of response in clinical trials is measuring bADAs and nADAs, which is then correlated to evidence of increased clearance (reductions in serum drug concentration) and loss of efficacy. Neutralizing ADAs to biological drug products differ from neutralizing antibodies to viruses/bacteria and traditional neutralization assays. Typically, nADAs are measured through the binding of a single epitope of the therapeutic product, whereas there may be multiple epitopes of the virus/bacteria that can result in the immunological neutralization of the infectious agent.

Thus, immunogenicity remains an important consideration in the development of biological drug products. Protein engineering strategies, including humanization, sequence optimization, and epitope modification, have reduced this immunogenic potential; however, some products are still associated with the development of ADAs. A further challenge that has long existed for biological drug products is that the development of ADAs is species-specific, and even non-human primates (NHPs) have not been able to routinely demonstrate translatable immunogenicity to biological drug products (5, 6). In addition, even though biosimilars no longer require clinical trials for most well-established monoclonal antibody products (7), there is still a need to investigate and address immunogenicity concerns that could arise during development and for novel biotherapeutics with limited clinical validation. These issues led us to conduct a “proof-of-concept” study to investigate the ability of NeoThy™ immune-humanized mice to produce meaningful ADA responses to biological drug products, rather than infectious agents. The rationale for this model is to support patient safety through hazard identification of immunogenicity and to provide an animal model that could support drug development (candidate drug selection), including biosimilar development.

The development of immune-humanized mice has advanced tremendously since the original CD34 and bone marrow–liver–thymus (BLT) models were first introduced (8, 9). There are many different strains of mice that can be used for humanization, including those engineered to express human cytokines (such as granulocyte-macrophage colony-stimulating factor (GM-CSF), IL-3, or IL-6) that aid in the development of myeloid lineage cells (10–15). There are also a range of techniques to enhance humanization that have been described (16), but as has been noted by many, the ability of immune-humanized mice to produce class-switched, antigen-specific antibodies has been deficient (17–20). NeoThy immune-humanized mice, first produced in 2018 (21), represent a further advance in humanization. They utilize either neonatal or pediatric thymus tissue, typically combined with hematopoietic stem cells (HSCs) from cord blood of the same donor, or combined with cord blood HSCs that have been human leukocyte antigen (HLA)-matched to several alleles of the thymus tissue.

In light of these advances, we sought to determine if NeoThy immune-humanized mice could generate antigen-specific ADA responses to therapeutic protein drug products known to have high rates of immunogenicity in patients (22–24). For this proof-of-concept study, we chose two drugs: infliximab, an anti-TNF monoclonal antibody used to treat autoimmune diseases, and interferon-β-1b, a protein therapeutic used to treat multiple sclerosis. According to Food and Drug Administration (FDA)-approved prescribing information, reported immunogenicity rates in treated patients range from approximately 10%–52% for antibodies to infliximab and 17%–45% for neutralizing antibodies to interferon-β-1b, depending on disease indication, treatment regimen, patient age, sampling time, and assay format (25–28). Given the high rates of immunogenicity to these products, it was likely that we would have at least one donor who would have positive responses. In addition to testing these products individually, we decided to test them in combination, as patients could be prescribed more than one biological drug product simultaneously. There are no studies to date that have examined whether there is an additive effect of immunogenic products.

The primary goal of this study was to determine whether NeoThy immune-humanized mice could produce antigen-specific ADAs to known immunogenic drug products. Our secondary goals were to characterize the immune phenotype of the mice and demonstrate that these mice can produce immunogenic responses to biological drug products by measuring adaptive immune activation, immunoglobulin class switching, and *ex vivo* proliferation and evaluating immune organ histology.

## 2 Materials and methods

### 2.1 Experimental design

A total of six surgery groups of mice were humanized by implanting neonatal or pediatric thymus tissue and injecting HSCs, with **Supplementary Figures 1** and **2** showing the humanization schematic and study design, respectively. Following humanization, all surgery groups entered the study and were assigned to treatment with interferon-β-1b (IFN-β), infliximab, a combination of IFN-β and infliximab, keyhole limpet hemocyanin (KLH), or saline (**Supplementary Figure 1**). The goal of the combination treatment was to determine if two immunogenic biological drugs would increase the likelihood of overall immunogenicity incidence as compared to each drug alone. Interim bleed/serum collection was completed, and all mice remaining in the study at 9 weeks were sacrificed to collect immune organs (lymph nodes, spleen, and bone marrow) for evaluation and tissues for histology as described below.

### 2.2 Animals

Five- to eight-week-old female NOD.Cg-*Prkdc^scid^ Il2rg^tm1Sug^*Tg(CMV-IL6)1-1Jic/JicTac mice (Taconic Biosciences, Germantown, NY, USA) were humanized either at the US FDA or by Taconic Biosciences. Mice humanized by Taconic Biosciences were shipped to the FDA for study testing 12–14 weeks after humanization surgery. Study mice were housed in their treatment groups in individually ventilated micro-isolator cages at the FDA animal facility, fed autoclaved LabDiet (St. Louis, MO, USA) formula #5K52, and provided autoclaved acidified water (pH ~ 2.9) *ad libitum*. Enrichment, including nestlets, egg carton, and an exercise wheel, was provided. Mice were monitored daily and weighed weekly. All procedures were conducted in Association for Assessment and Accreditation of Laboratory Animal Care (AAALAC)-accredited facilities and approved by the Institutional Animal Care and Use Committee of each institution.

### 2.3 Mouse humanization procedure

A total of six surgery groups were produced as shown in the schematic in **Supplementary Figure 1**. Individual groups of 18–30 mice were produced from fully or partially HLA-matched donor thymus/HSC, essentially yielding groups of mice that had the same human immune system for the noted donor group (**Supplementary Table 1**). Surgery groups 1–3 were humanized by the FDA, while groups 4–6 were humanized by Taconic Biosciences. NeoThy™ immune-humanized mice were generated as previously described (8); however, neonatal or pediatric thymus tissue was used, and no liver tissue was implanted. Briefly, on humanization day 0, surgery was performed to implant human thymus tissue underneath the kidney capsule. Approximately 2 weeks following surgery, mice were given 20–25 mg/kg busulfan intraperitoneally (i.p.) to ablate their bone marrow. Twenty-four hours later, they received a CD34^+^ HSC injection intravenously via the tail vein. Mice received 0.1 mg/kg, i.v., of anti-human CD2 (isolated and purified from hybridoma LO-CD2b, ATCC, Manassas, VA, USA), in two separate injections, to remove any passenger thymocytes or T cells in the HSC isolation. Beginning 9 to 12 weeks post-surgery, blood from the mice was evaluated for humanization every 4 weeks using antibodies against human and mouse immune receptors (**Supplementary Table 2**). Antibodies were purchased from BioLegend (San Diego, CA, USA), BD Biosciences (Franklin Lakes, NJ, USA), or Thermo Fisher (Waltham, MA, USA).

### 2.4 Donor tissue, hematopoietic stem cells, and study groups

For surgery groups 2 and 3, CD34^+^ HSCs were obtained from the same donor as the thymus; for groups 1, 4, 5, and 6, CD34^+^ HSCs were obtained from cord blood that matched at least three of six alleles for HLA-A, HLA-B, and HLA-DRB1 (**Supplementary Table 1**).

Thymus, femur, and cord blood were obtained from neonatal donors through the International Institute for the Advancement of Medicine (IIAM; Edison, NJ, USA), referred to as “neonatal”, and used for thymus and HSCs for groups 2 and 3, with thymus tissue only used for surgery groups 1, 5, and 6. CD34^+^ HSCs were isolated from the femur and cord blood following receipt. Other purified HSCs (groups 1, 4, 5, and 6) were received from a commercial vendor through Taconic Biosciences. Taconic obtained pediatric thymus from discarded medical waste tissue of neonatal cardiac surgery patients (University of Wisconsin) and used it for surgery group 4, labeled as “pediatric”. HLA genotyping for thymus and CD34 HSCs was completed when possible (**Supplementary Table 1**). A summary of the characteristics of all study mice, including the surgery group, donor source for thymus and HSC, sex, treatment assignment, and euthanasia date, is shown in **Supplementary Table 3**.

NeoThy immune-humanized mice were assigned to treatment groups based on their final humanization bleed data prior to entering the study. Major cell populations (T cells, B cells, and monocytes), markers of T-cell maturation/activation (CD45RA and CD45RO), and total humanization (based on the number of human CD45^+^ cells/μL blood) were used to assign mice into study groups. The goal was to ensure that the means of these populations were as equal as possible between groups at the start of the study.

### 2.5 Pharmaceuticals, treatment groups, and sampling intervals

Five treatment groups included infliximab (Remicade, Lot #24F045P1, Janssen Biotech, Inc., Horsham, PA), IFN-β-1b (Betaseron, NDC 50419-524-01, Bayer HealthCare Pharmaceuticals. Inc., Germany), infliximab and IFN-β, KLH (Cat. No. 374825–25 mg, EMD Millipore, Billerica, MA) with cholera toxin (Cat. No. C8052-0.5 mg, Sigma, St. Louis, MO), or saline (negative control). Within each surgery group (1–6), groups of four to six mice were randomized to receive 5 mg/kg infliximab weekly i.p., 5 μg/mouse IFN-β i.p. 5 days per week, infliximab and IFN-β at previously noted dose/route/schedule, 100 μL saline i.p. 5 days per week or 100 μg/mouse KLH on day 0, and 30 μg/mouse KLH on days 14 and 42, which included 1 μg/mouse cholera toxin i.p. The dose and treatment frequency for IFN-β and infliximab were selected to be comparable to those received in patients and were based on results of prior dose-range finding studies in immune-humanized mice at FDA (data not shown).

Normal pooled human serum and human ADA^+^ patient serum were purchased from BioIVT, Woodbury, NY. Mice were observed twice daily and weighed every other day. Mice were bled by tail vein to collect EDTA anti-coagulated blood (flow cytometry) and whole blood (for serum) at days 0, 21, 42, and 63 (necropsy). At study end, whole blood, EDTA anti-coagulated blood, bone marrow (BM), spleen (SP), and peripheral lymph nodes (LNs) were collected for aseptic processing.

Investigators were blinded to the identity of the treatment group during the processing of samples, the conduct of assays (e.g., flow cytometry, ELISA-based assays, and proliferation experiments), and the analysis of data.

In some instances, mice were terminated early due to weight loss or obvious illness. Early exits from the study are shown in **Supplementary Table 3** (euthanasia date) and in the survival curves. If the cause of illness (e.g., sepsis or tumor) was known, the mouse was excluded from flow cytometric analysis. Therefore, some tissue samples or assay endpoints had fewer than the maximum possible, based on study enrollment.

### 2.6 Bone marrow, spleen, and lymph node isolations

Splenocytes and BM cells were isolated and processed using standard techniques. To isolate the peripheral LN, the gross size was noted, and as much fat as possible was removed. LNs were minced using scissors in a sterile 100-μm cell strainer on a 50-mL conical tube on ice and then rinsed with cold media. After mincing, the rubber end of a sterile 3-mL syringe was used to mechanically disrupt the tissue, and then the strainer was rinsed with cold media. Mincing, disruption, and rinsing were completed a total of three times. Cells were then filtered using a 70-μm mesh and centrifuged at 4 °C for 5 minutes at 400 × *g*. The supernatant was removed by pipetting, and cells were resuspended in cold media for counting. SP, BM, and LN cells were counted using Trypan blue.

### 2.7 Flow cytometry

Flow cytometry was performed at all bleed dates (days 0, 21, and 42) and at necropsy. For peripheral blood mononuclear cells (PBMCs), EDTA anti-coagulated blood was collected, volume was quantified, and red blood cells were lysed using lysis buffer (BioLegend). Fc-receptor binding was blocked with purified anti-human Fcγ and anti-mouse CD16/32 Fcγ^+^ antibodies, followed by staining with the anti-human and anti-mouse antibodies (**Supplementary Table 2**) as previously described (29). Spleen, BM, and LN cells were blocked and stained following cell counting, as previously described (29). Samples were assessed immediately following staining on a five-laser Cytek Aurora (Cytek Biosciences, Fremont, CA, USA) flow cytometer. Definitions for each gated population are listed in **Supplementary Table 4**, with gating examples for PBMCs and SP shown in **Supplementary Figure 3**. All data were analyzed using FCS Express (*De Novo* Software, Pasadena, CA, USA).

### 2.8 Proliferation assay

Proliferation was assessed using CellTrace dyes (Thermo Fisher) and performed with blinding to sample identity. Briefly, 1 × 10^6^ spleen or LN cells were stained with CellTrace™ Blue in 15-mL conical tubes, according to the manufacturer’s protocol. Following staining, cells were added to a 48-well plate, with 2 × 10^5^ cells per condition (unstained/unstimulated, stained/unstimulated, 20 μg/mL Concanavalin A (ConA), 10 μg/mL IFN-β, 10 μg/mL infliximab, or 10 μg/mL KLH) and incubated for 66–72 hours at 37 °C in a 5% CO_2_ incubator. Following culture, cells were washed and run on a Cytek Aurora flow cytometer. Data were analyzed using FCS Express using the Fit → proliferation analysis tool. Division index was used to compare relative proliferation between *ex vivo* treatments and calculate fold increase over stained/unstimulated (baseline) for each mouse. Samples that had a greater than 1.5-fold increase over baseline were considered positive.

### 2.9 Immunoglobulin isotype assay

A commercial multiplex bead-based immunoassay (Millipore Sigma, Cat. No. HGAMMAG-301K, Burlington, MA) was used to measure total IgG_1–4_ and IgM, primarily according to the manufacturer’s instructions. Briefly, humanized mouse serum samples were thawed at room temperature, vortexed, and centrifuged at low speed to remove particulates. Pooled normal human serum (1:20,000 dilution) was a positive control, and experimental mouse serum samples were diluted 1:250 using assay buffer, with a total sample volume of 50 μL used for testing. All samples and standards were run in duplicate. The bead mix was sonicated before adding 25 μL to each well containing either sample or standard. The plate was sealed and incubated on a plate shaker at 500–600 rpm for 1 hour at room temperature (RT). Following incubation, beads were washed twice by placing the plate on a magnetic plate holder for 60 seconds, decanting the supernatant, and adding 200 μL of 1× Wash Buffer per well. After washing, 25 μL of anti-human κ and λ light chain detection antibody was added to each well, and the plate was incubated for 30 minutes under the same conditions, followed by the addition of 25 μL of Streptavidin-PE and another 30-minute incubation. The final wash was performed with 150 μL of sheath fluid per well, and the beads were resuspended by shaking for 5 minutes before data acquisition on a Bio-Plex^®^ 200 System based on Luminex xMAP^®^ technology (Bio-Rad Laboratories, Hercules, CA, USA). Data analysis was completed using the Bio-Plex Manager software, where duplicate readings for each standard were averaged, and a five-parameter logistic regression model was applied to generate the standard curve.

### 2.10 Anti-KLH IgM and IgG antibody assay

Semi-quantitative sandwich ELISA assays were developed to measure KLH-specific human IgM and IgG antibodies in immune-humanized mouse serum. A mouse IgG_1_ monoclonal anti-KLH antibody (BioLegend, Cat. No. 408402) was used for the generation of the standard curve. Non-study immune-humanized mice, treated with the same KLH regimen as used for this study, were screened to identify positive controls for anti-KLH IgM and anti-KLH IgG, as no human anti-KLH antibody serum was available. The identified anti-KLH IgM- and IgG-positive serum and pooled serum from naïve immune-humanized mice were used to evaluate matrix effects, select the optimal sample dilution, define the standard curve range, and assess assay sensitivity and positivity criteria. Background signal was assessed by testing pooled naïve immune-humanized mouse serum with the detection antibody alone across different matrix concentrations in each assay. Study samples were classified as positive only when the measured values exceeded those obtained from the naïve mice and were above the lower limit of quantitation (LLOQ).

The dynamic range of the standard curve was 0–32 ng/mL, with concentrations of 0, 0.5, 1, 2, 4, 8, 16, and 32 ng/mL and a linear range of 0.5–16 ng/mL. During assay development, a range of KLH-coating concentrations were tested to determine the concentration with the lowest background in the selected matrix using positive and negative controls.

Two 96-well microtiter plates (one for IgM detection and the other for IgG detection) were coated with 10 μg/mL/well of KLH (Thermo Scientific, Cat. No. 77600) in a final volume of 100 μL per well and incubated overnight at 4 °C. The following morning, the plates were washed five times with PBST (phosphate-buffered saline (PBS) containing 0.05% Tween 20). The plates were blocked with 300 μL of 5% bovine serum albumin (BSA) in PBST, followed by a two-hour incubation at room temperature. After blocking, the plates were washed five times with PBST. Standards were prepared in duplicate using murine anti-KLH antibody in 3% BSA in PBST. Immune-humanized mouse serum from the KLH- or saline-treated mice was prepared at a 1:100 dilution in PBST. Standards and samples were incubated for 2 hours at room temperature, followed by five washes with PBST. Following washes, the standard curve was detected using an horseradish peroxidase (HRP)-conjugated donkey anti-mouse IgG (H+L) antibody at 1:10,000 dilution (Jackson ImmunoResearch, Cat. No. 715-035-151, West Grove, PA). In the IgM detection plate, antibodies were detected using an HRP-conjugated goat F(ab′)_2_ anti-human IgM μ-chain antibody at a 1:3,000 dilution (SouthernBiotech, Cat. No. 2022-05, Birmingham, AL). In the IgG detection plate, antibodies were detected using an HRP-conjugated goat anti-human IgG Fc antibody at a 1:4,000 dilution (Invitrogen, Cat. No. A55745, Waltham, MA). After adding the detection antibody, plates were incubated for 1 hour at room temperature and washed five times with PBST. Signal was detected using 100 μL of QuantaBlu™ Fluorogenic Peroxidase Substrate (Thermo Fisher Scientific, Cat. No. 15169), followed by incubation for 10 minutes. The plates were immediately read using a Spark microplate reader at an excitation wavelength of 325 nm, with a range of 315–340 nm, and an emission wavelength of 420 nm, with a range of 370–470 nm.

Data processing and statistical analyses were completed using GraphPad Prism. Calibration curves were generated using a four-parameter logistic model, with acceptance defined as values within ±20% of the nominal concentrations and an R^2^ ≥ 0.990. Group comparisons were performed using one-way ANOVA followed by Tukey’s post-hoc test.

### 2.11 Anti-infliximab neutralizing antibody assay

A neutralizing ADA assay against the infliximab drug product was developed in sandwich ELISA format. Anti-infliximab antibody (HCA213, Bio-Rad) was used for the standard curve and spiked samples at the upper limit of quantification (ULOQ), high quality control (HQC), mid quality control (MQC), low quality control (LQC), and LLOQ levels. Control serum from two unique human donors with anti-infliximab antibodies [individuals treated with infliximab (Remicade) and then discontinued] was used to assess matrix effect, optimal dilution, standard curve range, positivity, and sensitivity. Immune-humanized mouse serum from pooled healthy non-study mice (control mice) was used to determine matrix effect, optimal dilution, sensitivity, and positivity. Background noise level was determined by testing control immune-humanized mouse serum with detection antibody alone, in a range of matrices at different concentrations. For study mice, only values exceeding those of control mice in the assay were considered positive. The dynamic range of the standard curve was 0–40 ng/mL, with concentrations of 0, 1.5, 2.5, 5, 10, 15, 25, and 40 ng/mL, with a linear range of 1.5–25 ng/mL. During assay development, drug coating concentrations of 0.25–0.75 μg/mL were tested to determine the concentration with the lowest background in the selected matrix using all positive and negative controls. This assay was not formally qualified; however, our Division’s Bioanalytical Group provided support in the development, validation, and interpretation of the assay.

A 96-well microtiter plate was coated overnight at 4 °C with 0.5 μg/mL of infliximab in a total volume of 100 μL/well. The following morning, plates were washed five times with PBST (PBS containing 0.05% Tween 20), followed by blocking with 300 μL of 5% BSA in PBST and incubation for 2 hours at RT. After blocking, plates were washed five times with PBST. Standards were prepared in duplicate using an anti-infliximab antibody HCA213 in 5% BSA in PBST with concentrations ranging from 0 to 40 ng/mL. Serum samples from infliximab- or saline-treated humanized mice were diluted (with PBST) 1:100, and human serum, known to contain anti-drug antibodies (BioIVT), as a positive control, was diluted 1:100 before incubation for 1 hour at RT, followed by five washes with PBST. Then, 100 μL of HRP-conjugated infliximab (diluted to 1.25 μg/mL in Tris-buffered saline with 0.05% Tween) was added to each well and incubated for 1 hour at RT.

Following incubation, plates were washed five times with PBST, and 100 μL of QuantaBlu™ Fluorogenic Peroxidase Substrate (Cat. No. 15169; Thermo Fisher Scientific) was added to each well. After a 10-minute incubation, fluorescence was measured using a Spark microplate reader (Tecan, Männedorf, Switzerland) with an excitation maximum of 325 nm (315–340 nm) and an emission maximum of 420 nm (370–470 nm). Data were analyzed using the GraphPad Prism software. Standard curves were fitted using a four-parameter logistic (4-PL) regression model. Acceptance criteria required standard concentrations to fall within ±20% of the expected values, with an R^2^ ≥ 0.990. For correlation analyses, simple linear regression with a best-fit line was applied. Statistical differences between treatment groups were evaluated using one-way ANOVA followed by Tukey’s multiple comparisons test.

### 2.12 Anti-IFN-β antibody assay

A semi-quantitative binding antibody assay, in sandwich ELISA format, was developed for the detection of binding ADAs to interferon-β1b. Due to the lack of a commercially available human anti-IFN-β antibody, we initially tested two different mouse anti-human IFN-β antibodies. While both antibodies worked in the assay, the antibody chosen had less background and was more sensitive. However, due to the use of the murine antibody for the standard curve, the determined concentrations are relative values and not exact.

Anti-IFN-β antibody (clone BB4, Bio-Rad) was used for the standard curve and spiked samples at the ULOQ, HQC, MQC, LQC, and LLOQ levels. Control serum from two unique human donors with anti-IFN-β antibodies [individuals treated with IFN-β (Betaseron) and then discontinued] was used to assess matrix effect, optimal dilution, standard curve range, positivity, and sensitivity. Immune-humanized mouse serum from pooled healthy non-study mice (control mice) was used to determine matrix effect, optimal dilution, sensitivity, and positivity. Background noise level was determined by testing control immune-humanized mouse serum with detection antibody alone, in a range of matrices at different concentrations. For study mice, only values exceeding those of control mice in the assay were considered positive. The dynamic range of the standard curve was 0–25 ng/mL, with concentrations of 0, 1.25, 2.5, 3.75, 5, 10, 17.5, and 25 ng/mL, with a linear range of 1.25–17.5 ng/mL. During assay development, drug coating concentrations of 5,000–50,000 IU were tested to determine the concentration with the lowest background in the selected matrix using all positive and negative controls. This assay was not formally qualified; however, our Division’s Bioanalytical Group provided support in the development, validation, and interpretation of the assay.

A 96-well microtiter plate was coated with 20,000 IU/well of IFN-β-1b drug product in a final volume of 100 μL per well and incubated overnight at 4 °C. The following morning, plates were washed five times with PBST (PBS containing 0.05% Tween 20). Plates were blocked with 300 μL of 5% BSA in PBST, followed by a 2-hour incubation at RT. After blocking, plates were washed five times with PBST. Standards were prepared in duplicate using a murine anti-IFN-β1b antibody (clone BB4, Bio-Rad) in 5% BSA in PBST with concentrations ranging from 0 to 25 ng/mL. Humanized mouse serum from IFN-β- or saline-treated groups was prepared at a 1:100 dilution in PBST. Standards/samples were incubated for 2 hours at RT, followed by five washes with PBST. Anti-IFN-β antibodies in humanized mouse serum or human positive control serum samples were detected by incubating plates for 30 minutes at room temperature with an HRP-goat anti-human IgG antibody at 1:10,000 in PBST (Jackson ImmunoResearch, Cat. No. 109-035-098). Standard curve anti-IFN-β antibodies were detected using an HRP-goat anti-human IgG antibody at 1:10,000 in PBST (Thermo Fisher Scientific, Cat. No. A16090). After washing the plates five times with PBST, signal was detected using 100 μL QuantaBlu™ Fluorogenic Peroxidase Substrate (Cat. No. 15169; Thermo Fisher) with incubation for 10 minutes, after which plates were immediately read using a Spark microplate reader at an excitation wavelength of 325 nm (315–340 nm) and an emission wavelength of 420 nm (370–470 nm).

Data processing and statistical analyses were conducted using GraphPad Prism. Calibration curves were generated using a 4-PL model, with acceptance defined as values within ±20% of nominal concentrations and an R^2^ ≥ 0.990. Group comparisons were performed using one-way ANOVA with Tukey’s post-hoc test.

### 2.13 Histological evaluation

At necropsy, gross pathology observations were recorded, e.g., if normal or if gross lesions were present. Femur and sternum were initially fixed in Formical-4 (StatLab, McKinney, TX, USA) for 24 hours and then transferred to 10% neutral buffered formalin (NBF). All other tissues were placed in NBF at necropsy for fixation prior to processing. Tissues were processed through a series of graded alcohols, xylene, and paraffin (H&E) using an ASP300 S tissue processor (Leica Biosystems, Buffalo Grove, IL, USA). They were embedded in paraffin blocks, sectioned into 4-μm slices, mounted on glass slides, and stained with H&E (SelecTech, Leica Biosystems). Blind evaluation of slides was completed via light microscopy by a board-certified veterinary pathologist.

### 2.14 Statistical analysis

GraphPad Prism 10 was used for data analysis and graph formatting. GraphPad InStat, version 3.10, supported selected statistical analyses. Data are presented as mean ± SD or SEM, as noted in the figure legends. One-way or two-way ANOVA with an appropriate multiple comparisons test was used to compare differences among treatment groups, with specific tests noted in figure legends. Statistical significance was indicated as *p < 0.01, **p < 0.001, and ***p < 0.0001.

## 3 Results

### 3.1 NeoThy™ immune-humanized mice produce all major immune cell populations

An important consideration in evaluating immune-humanized mouse studies is the overall humanization of the mice. Mice should be sufficiently humanized to produce immune responses and assigned to treatment groups based on equivalent humanization so that results can be compared. For the assessment of blood, we typically use human CD45^+^ cells per μL of blood rather than percentages; however, both are shown for completeness.

**Figure 1:**
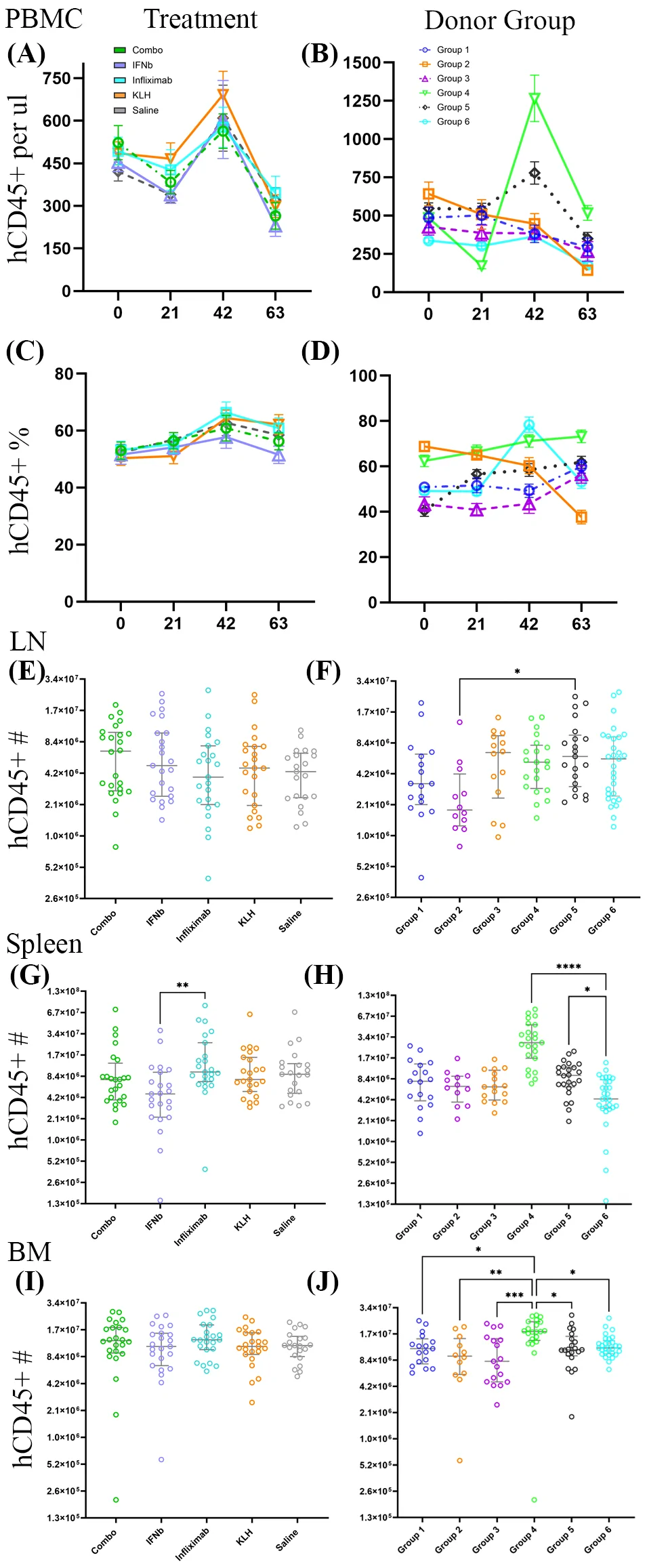
Quantification of human CD45^+^ leukocyte reconstitution in NeoThy immune-humanized mice. PBMCs are shown by treatment **(A, C)** and donor **(B, D)** based on cells per μL blood **(A, B)** and percentage **(C, D)** of human CD45^+^ cells. Absolute cell yields of human CD45^+^ cells in lymph nodes **(E, F)**, spleen **(G, H)**, and bone marrow **(I, J)** at necropsy are presented by treatment group and donor, respectively. Treatment groups (left column) are noted as green (combo), purple (IFN-β), light blue (infliximab), orange (KLH), and gray (saline). Donor groups (right column) are noted as blue (donor 1), orange (donor 2), purple (donor 3), green (donor 4), black (donor 5), and light blue (donor 6). Data points represent individual group means for PBMC and individual mice for LN, SP, and BM. Total n for treatment groups at necropsy (combo, infliximab, IFNβ, KLH, and saline were 24, 23, 23, 21, and 21, respectively). Total n for donor groups 1–6 at necropsy were 17, 12, 14, 21, 21, and 27. Statistics were calculated using a two-way ANOVA with Tukey’s multiple comparisons post-test for PBMC and Kruskal–Wallis ANOVA with Dunn’s multiple comparisons post-test for tissues. Mean values ± SEM are shown for PBMC, with box and whisker plots showing the median and interquartile range for tissues. Statistical differences are shown with an asterisk if p < 0.05. PBMC, peripheral blood mononuclear cell; LN, lymph node; SP, spleen; BM, bone marrow; KLH, keyhole limpet hemocyanin.

**Figure 2:**
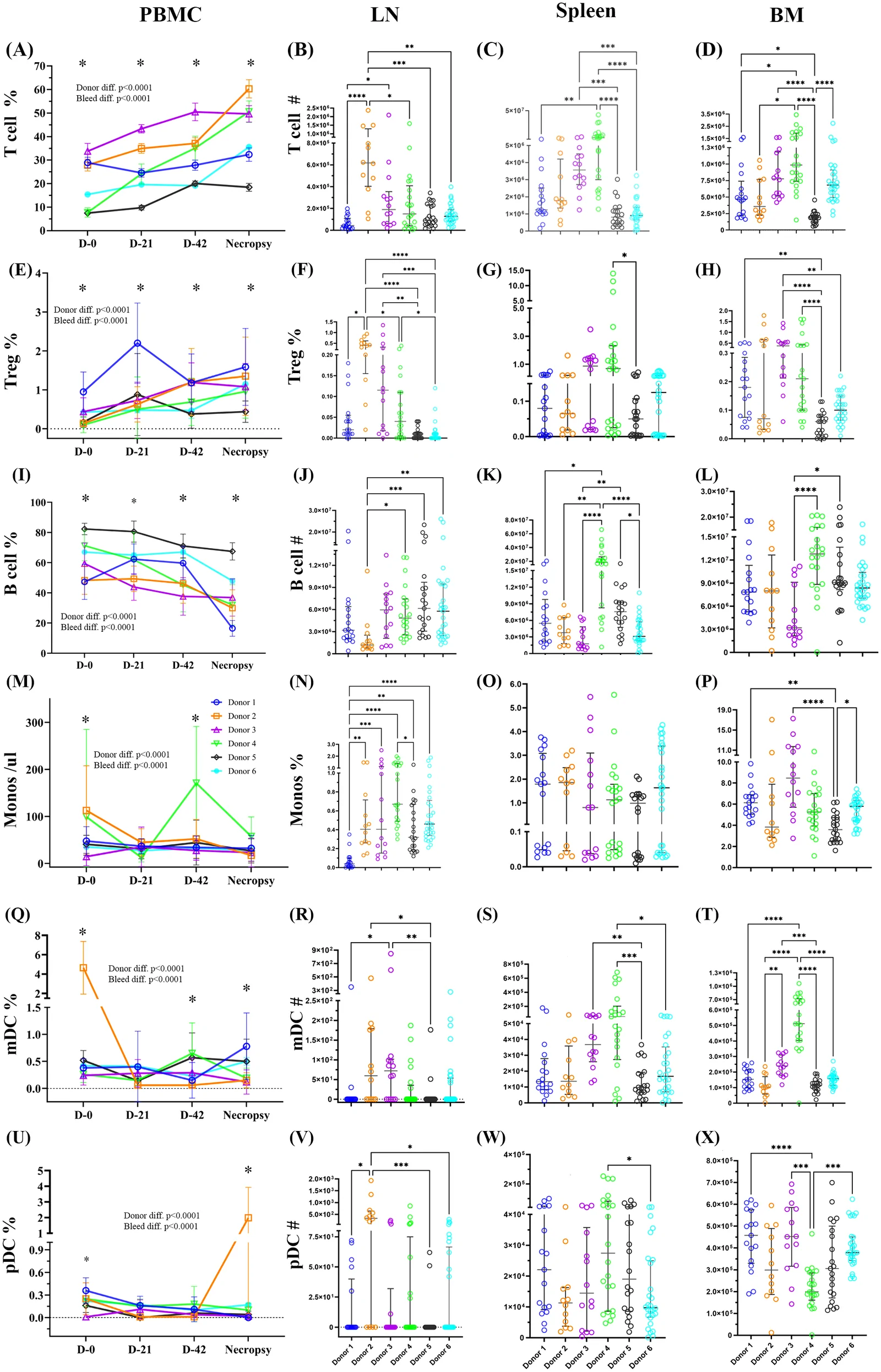
Distribution of human immune cell subsets in PBMC and tissues by donor. Major cell populations shown are T cells [percent in PBMC **(A)** and absolute number for LN **(B)**, SP **(C)**, and BM **(D)**], percent Tregs **(E–H)**, B cells [percent in PBMC **(I)** and absolute number for LN **(J)**, SP **(K)**, and BM **(L)**], monocytes [cells per μL blood in PBMC **(M)**, percent for LN **(N)**, SP **(O)**, and BM **(P)**], mDCs [percent in PBMC **(Q)** and absolute number for LN **(R)**, SP **(S)**, and BM **(T)**], and pDCs [percent in PBMC **(U)** and absolute number for LN **(V)**, SP **(W)**, and BM **(X)**]. Donor groups are noted as blue (donor 1), orange (donor 2), purple (donor 3), green (donor 4), black (donor 5), and light blue (donor 6). Total n of donor groups 1–6 at necropsy were 17, 12, 14, 21, 21, and 27, respectively. For PBMC, data points represent individual group means. Individual mice are shown for LN, SP, and BM. Mean values ± SEM are shown for PBMC, with box and whisker plots showing the median and interquartile range for tissues. Statistics were calculated using two-way ANOVA with Tukey’s multiple comparisons post-test for PBMC and Kruskal–Wallis ANOVA with Dunn’s multiple comparisons post-test for LN, SP, and BM. For two-way ANOVA, overall differences based on treatment cohort and bleed date are noted in the graph, while specific differences between cohorts at a timepoint are noted with an asterisk if p < 0.05. PBMC, peripheral blood mononuclear cell; LN, lymph node; SP, spleen; BM, bone marrow; mDCs, monocytic dendritic cells; pDCs, plasmacytoid dendritic cells.

**Figure 3:**
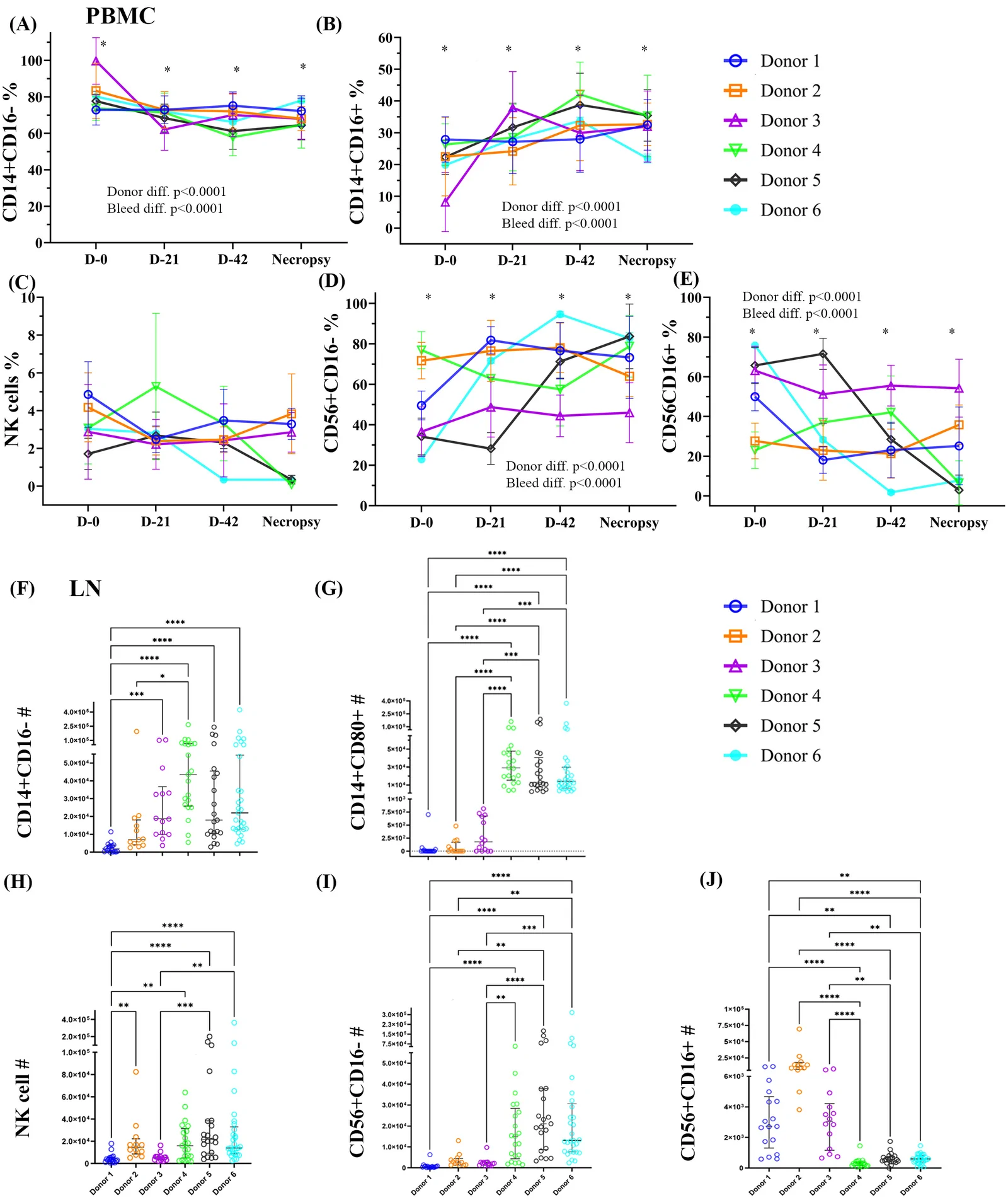
Distribution of monocyte and NK cell subsets in PBMC and LN based on donors. For PBMC, percent CD16^+^ or CD16^−^ monocytes are shown **(A, B)**, with total NK cell percentage **(C)**, and percentage NK cells expressing CD16^+^ and CD16^−^ **(D, E)** over time. LN absolute cell counts are shown for CD16^−^ monocytes **(F)** and CD14^+^CD80^+^ monocytes **(G)**, with absolute cell counts for NK cells **(H)**, CD56^+^CD16^−^ **(I)**, and CD56^−^CD16^+^ **(J)**. Donor groups are noted as blue (donor 1), orange (donor 2), purple (donor 3), green (donor 4), black (donor 5), and light blue (donor 6). Data points represent individual group means for PBMC and individual treated mice for LN. Total n of donor groups 1–6 at necropsy were 17, 12, 14, 21, 21, and 27, respectively. Mean values ± SEM are shown for PBMC, with box and whisker plots showing the median and interquartile range for the lymph node. Statistics were calculated using two-way ANOVA with Tukey’s multiple comparisons post-test for PBMC and Kruskal–Wallis ANOVA with Dunn’s multiple comparisons post-test for LN. For two-way ANOVA, overall differences based on treatment cohort and bleed date are noted in the graph, while specific differences between cohorts at a timepoint are noted with an asterisk if p < 0.05. PBMC, peripheral blood mononuclear cell; LN, lymph node.

All major immune cell populations were evaluated in peripheral blood (PBMCs) over time and in peripheral LN, SP, and BM at necropsy (**Figures 1**–**3**). All mice in the study were adequately humanized to evaluate their immune populations, with human CD45^+^ cells averaging 421–523 leukocytes/μL blood at study start and averages ranging from 230 to 692 leukocytes/μL blood during the study (**Figure 1A**). The percentage of human CD45^+^ cells averaged 52% for all treatment groups and ranged from 51% to 66% during the study (**Figure 1C**). Mean yield for LNs ranged from 4.65 × 10^6^ to 7.66 × 10^6^ (**Figure 1E**), SP ranged from 7.22 × 10^6^ to 18.7 × 10^6^ (**Figure 1G**), and BM ranged from 11.66 × 10^6^ to 14.26 × 10^6^ (**Figure 1I**), with the spleen having significant differences in yield between treatments, primarily a result of higher yields for donor 4 (**Figure 1H**). CD45 humanization is shown by the donor group in **Figures 1B, D, F, H, J** and showed that some significant differences in yield were due to differences between donors, rather than treatments. Despite the donor-associated differences, NeoThy immune-humanized mice achieved sufficient humanization so that they would be capable of responding to immune stimulation.

An important consideration in humanization is that all necessary cell populations, including innate and adaptive human leukocytes, are present to enable cellular crosstalk and the production of appropriate cytokines. We evaluated all isolated immune populations, including PBMCs, LNs, SP, and BM, for the presence of these immune subsets. All donors showed populations of T cells (**Figures 2A–D**), Tregs (**Figures 2E–H**), B cells (**Figures 2I–L**), monocytes (**Figures 2M–P**), monocytic dendritic cells (mDCs; **Figures 2Q–T**), and plasmacytoid dendritic cells (pDCs; **Figures 2U–X**).

Every cellular population had at least one significant difference noted between donors and tissues, except for monocytes in the spleen. Some donors had very clear differences, as compared to all others, in specific tissues. Examples include donor 2 with the most T cells and Tregs and the least B cells in LNs, donor 1 with the lowest number of monocytes in LNs, and donor 4 with the greatest number of mDCs in SP and BM. The variety of differences between donors contributed to some of the observed differences in these populations when evaluating them based on treatment (**Supplementary Figure 4**) and the lack of statistical differences in many treatment populations, as all donor groups were divided equally among treatments. Notably, the KLH- and saline-treated groups had significant differences in Treg and monocyte percentages in the spleen (**Supplementary Figures 4G, O**) versus the tested biologics, which were uniform in the treatment group, suggesting a treatment-related effect.

Additional cellular populations, including monocyte subsets and NK cells, were evaluated in PBMCs and LNs in **Figure 3**. Regardless of donor (**Figure 3A**) or treatment (**Supplementary Figure 5A**), classical monocytes (CD14^+^CD16^−^) constitute the majority of monocytes in PBMCs at study start, with increases to non-classical monocytes (CD14^+^CD16^+^, **Figure 3B**; **Supplementary Figure 5B**) occurring over time, particularly in KLH- and saline-treated groups. NK cells in PBMCs varied significantly between donors (**Figure 3C**), with donors 1–3 remaining consistent over the course of the study, while those in donors 4–6 decreased by the end of the study. Due to the distribution of mice from each donor among treatments, no differences based on treatment were observed, other than those due to timepoint (**Supplementary Figure 5C**). In LNs, greater than 95% of monocytes at necropsy were CD14^+^CD16^−^ (**Figure 3F**; **Supplementary Figure 5F**), with donors 1 and 2 having statistically fewer absolute numbers of monocytes than other donors. There were significantly more activated monocytes for donors 4–6, as compared to donors 1–3, as shown by increased CD80 expression **Figure 3G**. LN NK cells also showed significant differences between donors (**Figure 3H**), with a donor-dependent, treatment-independent effect on LN NK cells (**Supplementary Figure 5H**). There was a clear pattern of donor-based differences for NK cell CD16 expression (**Figures 3I, J**) with donors 1–3 showing predominantly CD56^+^CD16^+^ cells and donors 4–6 having primarily CD56^+^CD16^−^ NK cells. No treatment-based differences were noted (**Supplementary Figure 5I, J**).

Overall, these immune subsets showed great variability due to unique donor immune systems; however, all donors had sufficient immune cells of each cell type to produce immune responses to the test articles.

### 3.2 Treatment with immunogenic therapeutic proteins induces activation in T cells and B cells

The selection of biological drug products was based on well-established knowledge of the biotherapeutics induction of nADAs in patients. In addition, we had previously conducted pilot studies to evaluate a range of doses, consistent with therapeutic doses, to determine the optimal dose and treatment regimen for immune-humanized mice. Each drug was given to groups of mice as a single drug treatment or as a combination of both products. The decision to treat mice with both immunogenic biotherapeutics stemmed from the question of whether there could be an increased likelihood of immunogenicity if a patient was being treated with more than one biologic, as this is not typically addressed in clinical trials.

**Figure 4:**
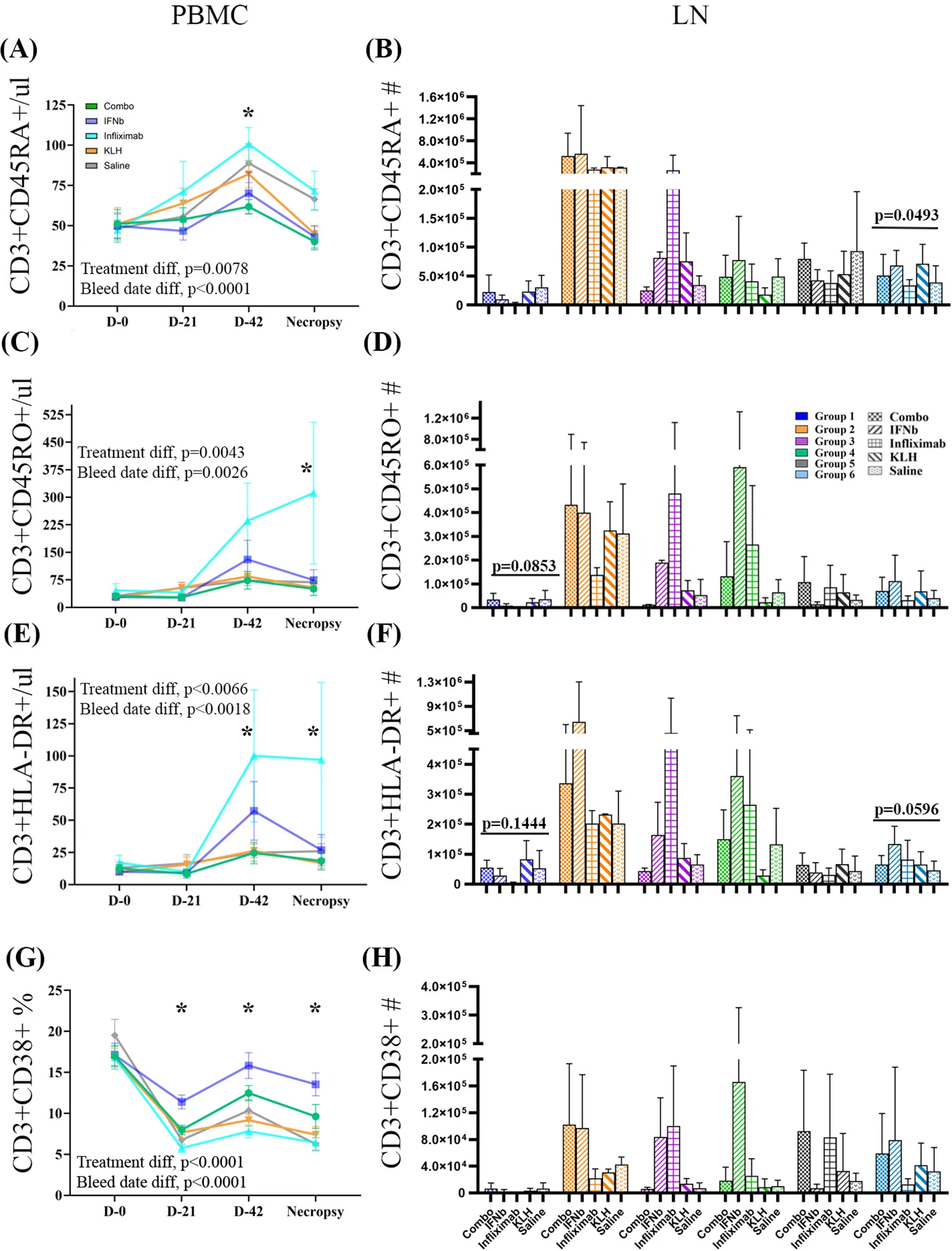
T-cell activation in PBMC and LN of NeoThy immune-humanized mice during the study. T-cell maturation/activation was evaluated in PBMC by treatment group using CD45RA^+^ [**(A)**; cells/μL blood), CD45RO^+^ **(C)**; cells/μL blood], HLA-DR^+^ [**(E)**; cells/μL blood], and CD38^+^ **(G)**; percentage]. PBMC treatment cohorts are noted as combo (green), infliximab (light blue), IFN-β (purple), KLH (orange), and saline control (gray). The same populations were evaluated in LN, showing absolute cell numbers for CD45RA^+^ **(B)**, CD45RO^+^ **(D)**, HLA-DR^+^ **(F)**, and CD38^+^ **(H)**. Donor groups for LN are noted as blue (group 1), orange (group 2), purple (group 3), green (group 4), black (group 5), and light blue (group 6). LN treatments in these donor groups are shown with their pattern in the graph below panel **(H)** Data points represent individual group means for PBMC, and treatment means for LN. Total n based on treatment cohorts (combo, infliximab, IFN-β, KLH, and saline) at necropsy (24, 23, 23, 21, and 21, respectively). Total n for donor groups 1–6 at necropsy were 17, 12, 14, 21, 21, and 27, respectively. Data are shown as mean ± SD using a two-way ANOVA with Tukey’s multiple comparisons post-test for PBMC and Kruskal–Wallis ANOVA with Dunn’s multiple comparisons post-test for tissues. For two-way ANOVA, overall differences based on donor group and bleed date are noted in the graph, while specific differences between groups at a timepoint are noted with an asterisk if p < 0.05. PBMC, peripheral blood mononuclear cell; LN, lymph node; KLH, keyhole limpet hemocyanin.

To determine if treatment with immunogenic therapeutic proteins such as infliximab and IFN-β-1b could induce immunogenicity in NeoThy immune-humanized mice, we evaluated a range of maturation and activation markers in T and B cells. Naïve T cells (CD3^+^CD45RA^+^) were assessed in PBMCs (**Figure 4A**), and all treatment groups had similar numbers of CD45RA^+^ T cells/μL at day 0; diverging from each other over time, peak differences were noted at day 42, where infliximab- versus combo-treated mice were statistically different. Activated T cells/μL in PBMCs (CD3^+^CD45RO^+^; **Figure 4C**) were low and similar at study start, with the number for infliximab increasing at day 42 and peaking at necropsy with significant differences versus every other group. While there were no differences at study start for CD45RA^+^ and CD45RO^+^ T cells by treatment, significant differences were evident based on donor at D0, D21, and D42 for CD45RA and at D42 and necropsy for CD45RO (**Supplementary Figures 6A, D**).

LN CD3^+^CD45RA^+^ T-cell absolute numbers are shown by donor (donors 1–6; left to right), comparing treatments (combo, IFN-β, Inflix, KLH, and saline; from left to right) (**Figure 4B**), demonstrating that donor-specific differences in response are present. Two-way ANOVA showed differences between treatments for donor 6, but the remaining donors/treatments generally had consistent numbers of naïve T cells present within their donor group. A similar pattern was observed for CD3^+^CD45RO^+^ T-cell absolute numbers (**Figure 4D**); however, donors 3 and 4 showed a trend of increased activation in IFN-β-treated mice. Notably, donor groups 1, 5, and 6, which had the same thymus HLA, and group 1, which shared the donor HSCs present in donors 5 and 6, showed similar response patterns, indicating the reproducibility of results. In the spleen (**Supplementary Figure 7A, B**), similar donor-based differences were observed for both CD45RA^+^ and CD45RO^+^, with donor 4 consistently showing increased CD45RO expression as compared to other donors for drug treatments and saline.

T cells expressing HLA-DR in PBMCs (**Figure 4E**) increased at day 42 and necropsy for infliximab-treated mice as compared to other groups, which appeared to be driven by donor 4 (**Supplementary Figure 6G**). LN CD3^+^HLA-DR^+^ absolute cell numbers (**Figure 4F**) showed a similar overall donor responsiveness pattern, with similar trends in treatment response for donors 1 and 6, and a trend of increased expression in IFN-β- and infliximab-treated mice in donors 2–4.

Interestingly, at the start of the study, all PBMC treatment groups had 15%–20% of T cells expressing CD38 (**Figure 4G**), which decreased by day 21, then rebounded for combo and IFN-β at day 42, and remained elevated at study end. In LNs, CD38^+^ expression (**Figure 4H**) was most noticeable for mice treated with the combination of IFN-β and infliximab for donors 2–6 but was not statistically significant.

Thus, T-cell activation, while influenced by the donor, suggests that donors 3 and 4 showed a trend of activation with IFN-β and infliximab treatments.

**Figure 5:**
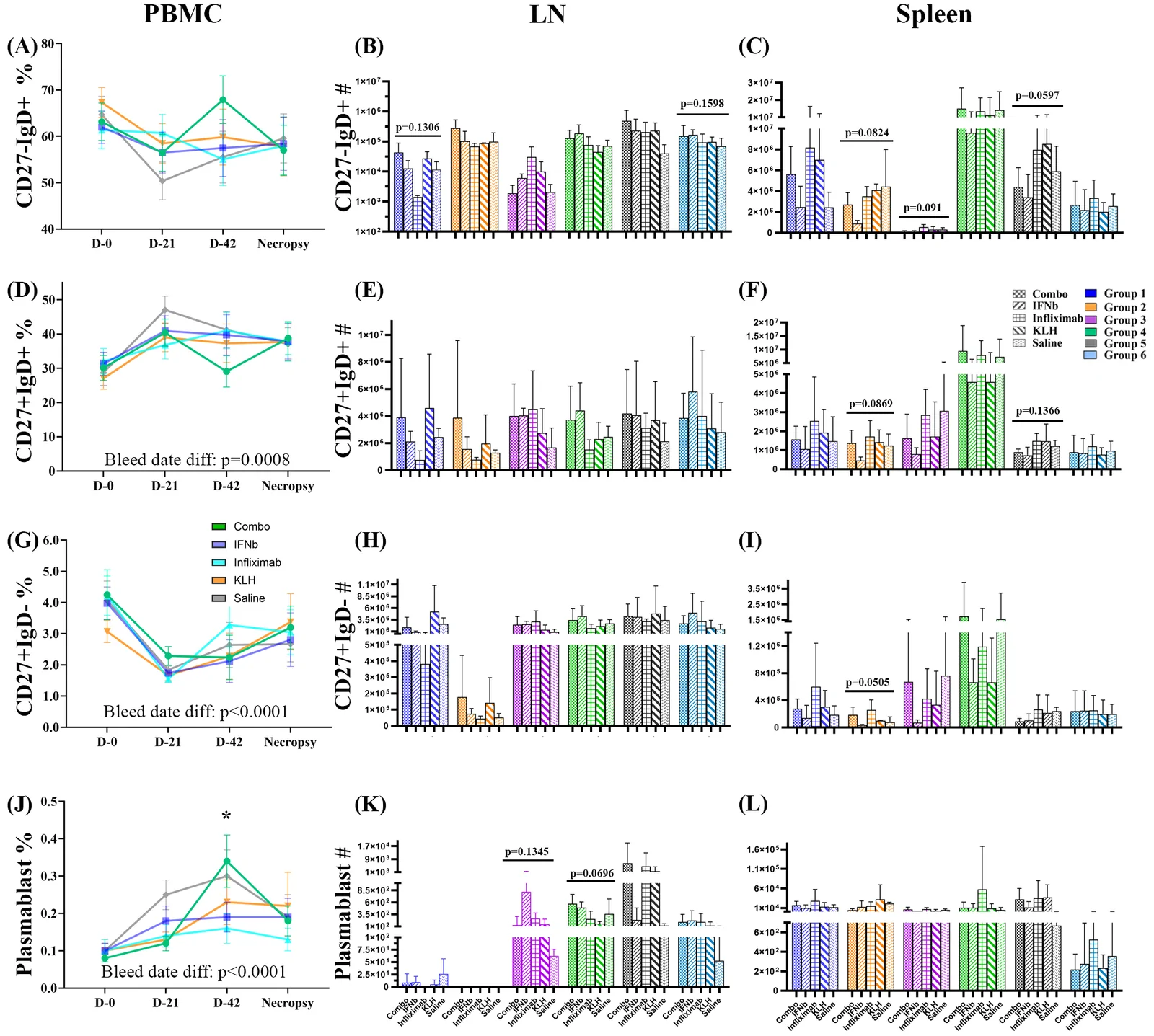
B-cell activation in blood and tissues. B-cell maturation/class switching was evaluated in PBMC (percentage of B cells), LN, and spleen (absolute cell number) for CD27^−^IgD^+^ (**A–C**; naïve), CD27^+^IgD^+^ [**(D–F)**; activated), and CD27^+^IgD^−^ **(G–I)**; class-switched] expression and the presence of plasmablasts **(J–L)**. PBMC treatment cohorts are noted as combo (green), infliximab (light blue), IFN-β (purple), KLH (orange), and saline control (gray). Donor groups are noted as blue (group 1), orange (group 2), purple (group 3), green (group 4), black (group 5), and light blue (group 6). LN and SP treatments for each donor are shown with their pattern in the graph below panels **(K, L)**. Data points represent individual group means for PBMC, and treatment means for tissues. Total n based on treatment cohorts (combo, infliximab, IFN-β, KLH, and saline) at necropsy (24, 23, 23, 21, and 21, respectively). Total n for donor groups 1–6 at necropsy were 17, 12, 14, 21, 21, and 27, respectively. Data are shown as mean ± SD using a two-way ANOVA with Tukey’s multiple comparisons post-test for PBMC and Kruskal–Wallis ANOVA with Dunn’s multiple comparisons post-test for tissues. For two-way ANOVA, overall differences based on donor group and bleed date are noted in the graph, while specific differences between groups at a timepoint are noted with an asterisk if p < 0.05. PBMC, peripheral blood mononuclear cell; LN, lymph node; KLH, keyhole limpet hemocyanin; SP, spleen.

B-cell subsets for PBMCs, LNs, and SP were evaluated for class switching using CD27^−^IgD^+^ (naïve; **Figures 5A–C**) and CD27^+^IgD^+^ (activated; **Figures 5D–F**) and class-switched using CD27^+^IgD^−^ (**Figures 5G–I**). In PBMCs, differences were noted between bleed dates in the percentage of activated and class-switched B cells (**Figures 5A, D, G**), with the majority of changes observed based on donor (**Supplementary Figures 6B, E, H**), particularly donor 3, where naïve cells decreased and activated/switched cells increased during the study. For LNs, no statistical differences were observed within donors based on treatment for any of the B-cell subsets (**Figures 5B, E, H**), potentially due to high variability combined with small group sizes. In the spleen, lower variability allowed for clear differences to be observed in cell number by donor. Donors 2, 3, and 5 had trends or significant differences between treatments for naïve, activated, and/or switched B cells. BM also exhibited significant donor variability in B-cell numbers based on activation status, with donor 2 having significantly more activated and class-switched B cells for treated versus control mice (**Supplementary Figures 6C, F, I**).

The percentage of plasmablasts (defined as B cell^+^/CD24^−^CD38hi^+^) in PBMCs was similar among treatments at day 0 and increased for most groups thereafter, with the combo group showing the greatest proportion in blood (**Figure 5J**), with significant differences noted by donor at all timepoints except day 42 (**Supplementary Figure 6J**). LN plasmablasts varied substantially by donor (**Figure 5K**), with a few detected in donors 1 and 2. All other groups had substantial numbers of plasmablasts, with trends of increased numbers in treated groups. Variability between donors was also observed in the spleen (**Figure 5L**) and BM (**Supplementary Figure 6L**), with fewer plasmablasts observed in some saline groups as compared to controls; however, none were significant.

Overall, the data showed that all donors were capable of B-cell class switching and producing plasmablasts, with some effects appearing to be linked to treatment.

### 3.3 Immunoglobulin class switching occurs in NeoThy immune-humanized mice

**Figure 6:**
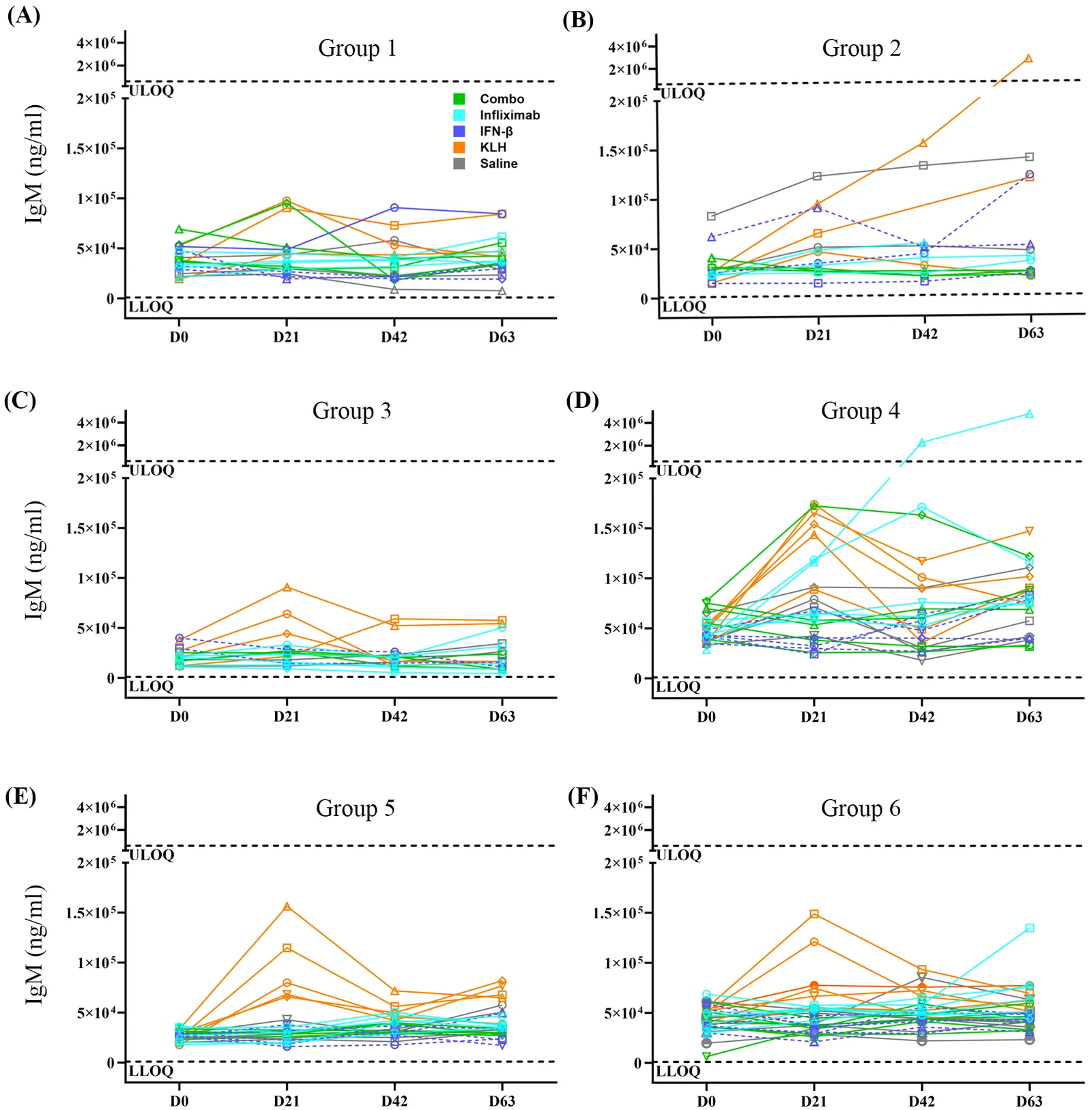
Total human IgM antibody levels by donor and treatment groups. IgM concentrations (ng/mL) were measured for each mouse in serum at days 0, 21, 42, and necropsy. Total mice tested were n = 127 (with n = 18, 14, 16, 24, 25, and 30, in panels **A-F** for donors 1–6, respectively) and by treatment with infliximab (n = 28), IFN-β (n = 25), combination (n = 26), KLH (n = 26), or saline (n = 22). Treatment groups are noted as combo (green), infliximab (light blue), IFN-β (purple), KLH (orange), and saline control (gray). Data are presented as antibody concentrations (ng/mL) over time. The lower and upper horizontal dashed lines denote the lower limit of quantification (LLOQ) and upper limit of quantification (ULOQ), respectively. Some individual data lines overlap, making them visually indistinguishable in the figure. KLH, keyhole limpet hemocyanin.

**Figure 7:**
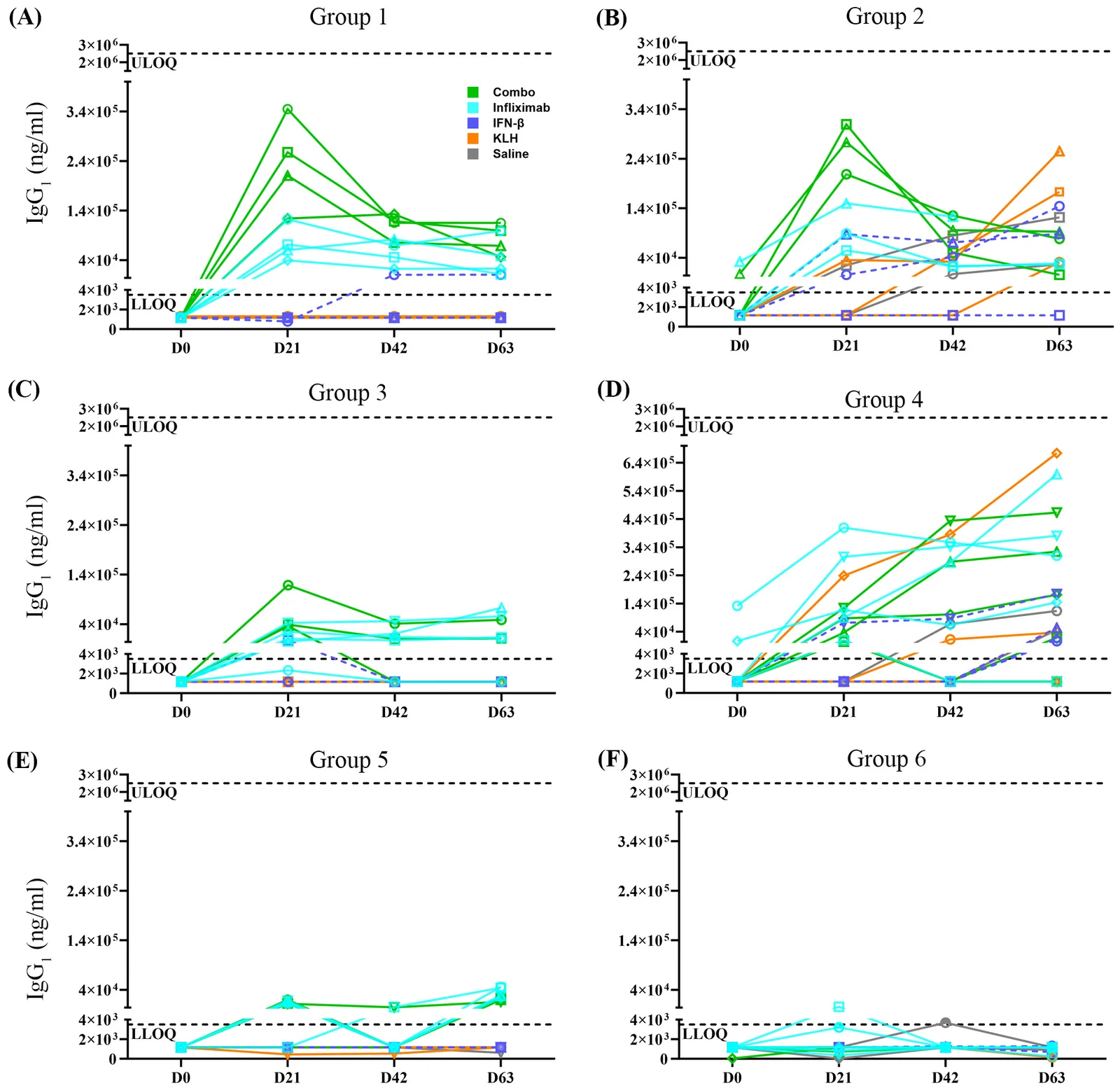
Total human IgG_1_ antibody levels by donor and treatment group. IgG_1_ concentrations (ng/mL) were measured for each mouse in serum at days 0, 21, 42, and necropsy. Total mice tested were n = 127 (with n = 18, 14, 16, 24, 25, and 30, in panels **A-F**, for donors 1–6, respectively) and by treatment with infliximab (n = 28), IFN-β (n = 25), combination (n = 26), KLH (n = 26), or saline (n = 22). Treatment groups are noted as combo (green), infliximab (light blue), IFN-β (purple), KLH (orange), and saline control (gray). Data are presented as antibody concentrations (ng/mL) over time. The lower and upper horizontal dashed lines denote the lower limit of quantification (LLOQ) and upper limit of quantification (ULOQ), respectively; individual data lines falling at or below the LLOQ may overlap and are visually indistinguishable. KLH, keyhole limpet hemocyanin.

All mice, regardless of treatment group or donor, had detectable levels of IgM (**Figures 6A–F**). Many KLH-treated mice had increased IgM at day 21, which typically fell by day 42. In general, very few infliximab- or IFN-β-treated mice showed significant increases in IgM, with the exception of a few mice in group 4. In contrast, many IFN-β-, infliximab-, and combo-treated mice had noticeable increases in IgG_1_ at day 21 (**Figures 7A–F**) with differences noted based on the donor. These increases leveled off for mice in groups 1, 2, and 3, whereas many in group 4 continued to show increasing levels until necropsy. Very few KLH-treated mice had increased IgG_1_, with the exception of a few mice in groups 2 and 4. The levels of IgG_2_ (**Supplementary Figures 8A, C, E**) and IgG_4_ (**Supplementary Figures 8B, D, F**) are shown for groups 1, 2, and 4, which showed significant levels of class switching. Only a few mice in group 1 demonstrated IgG_2_ or IgG_4_ class switching, whereas many mice in groups 2 and 4 class-switched to IgG_2_ or IgG_4_, regardless of study treatment. The same pattern was observed for mice in groups 1–4, for IgG_3_ (**Supplementary Figures 9A–D**), in which most mice in groups 2 and 4 class-switched to IgG_3_, whereas groups 1 and 3 had levels closer to the LLOQ, with only a few above it.

As expected, all NeoThy immune-humanized mice produced IgM. Donors 1–4 had significant class switching for IgG_1_ in most drug-treated groups, with donors 2 and 4 having additional class switching to IgG_2–4_, showing that while the donor dictates the magnitude of IgG class switching, clear treatment effects can be observed.

### 3.4 Lymph node and thymus histology support the development of class-switched antibodies

**Figure 8:**
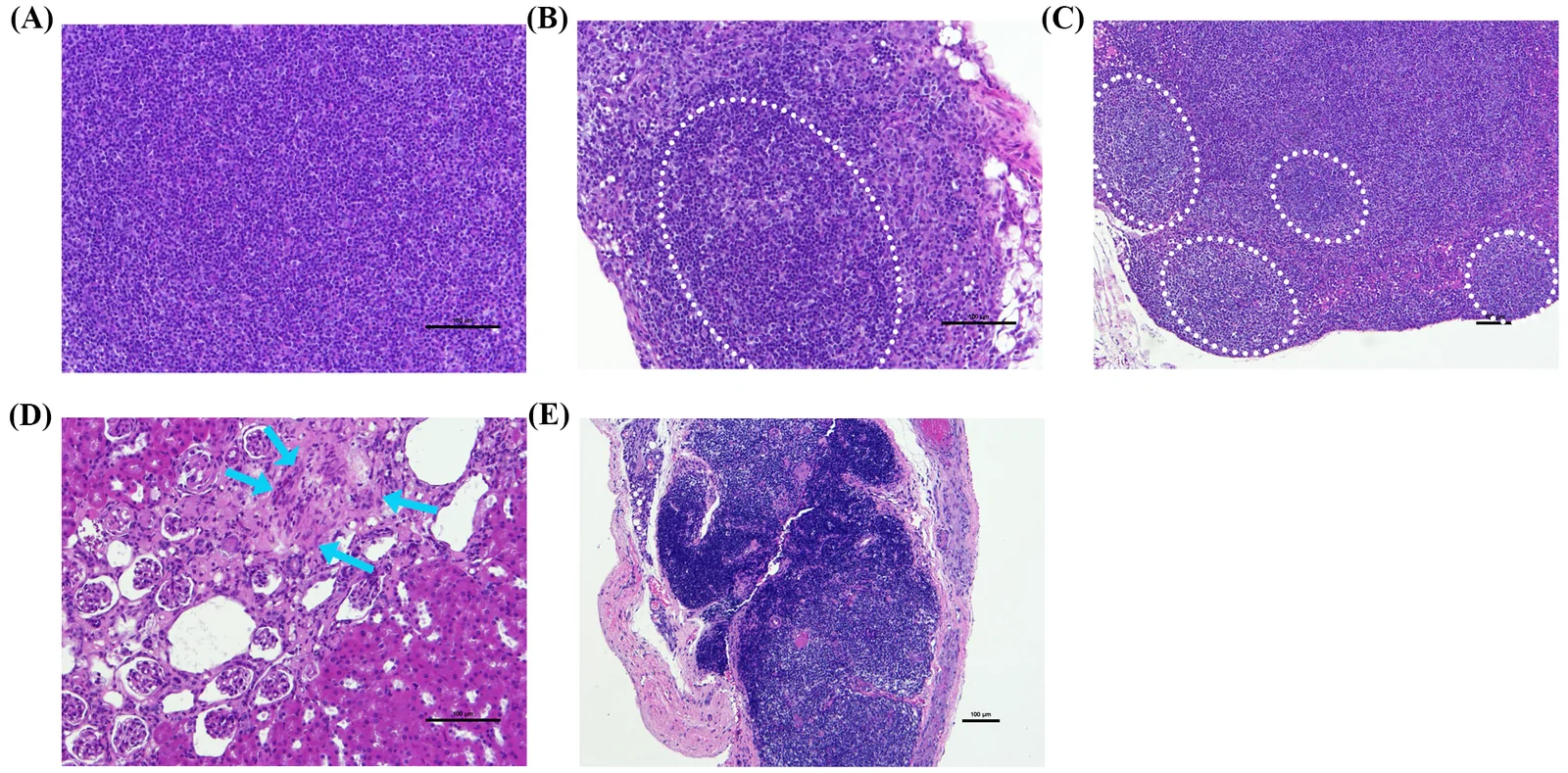
Histology of lymphoid and hematopoietic tissues in NeoThy immune-humanized mice. Representative hematoxylin and eosin (H&E)-stained sections of mesenteric lymph node (MLN) [**A** (×20), **B** (×20), and **C** (×10)] and thymus [**D** (×20) and **E** (×10)] collected at necropsy. White dashed lines **(B, C)** show the outer border of several germinal centers/B-cell follicles. Surgical scar **(D)** noted with turquoise arrows. Scale bars are indicated in each panel.

To better understand the immune responses that we observed, histological evaluation was performed by a board-certified pathologist who was blinded to donor and treatment assignments. Mesenteric lymph nodes (MLNs) and thymus (grafted onto the kidney) are shown in **Figure 8**, with the spleen and bone marrow (femur/sternum) discussed in the **Supplementary Results**/**Supplementary Figure 10**.

MLNs consistently displayed a homogeneous mixture of lymphocytes and histiocytes with no granulomas (**Figure 8A**). Classic lymph node architecture was not present; however, lymphoid follicles containing occasional giant cells were observed (**Figures 8B, C**), with plasma cells found in the medullary cords and sinuses (**Figures 8B, C**), consistent with the development of class-switched antibodies. While thymus tissue was typically observed to be present underneath the kidney capsule at necropsy, it was grossly small. Consistent with the finding of a small thymus, it was difficult to obtain a section containing the thymus to evaluate histologically. Most frequently, the surgical scar (**Figure 8D**) was observed. Thymus tissue that could be evaluated showed typical architecture (**Figure 8E**), with or without thymic cortex and medulla present, depending on the section evaluated. Proper architecture of the thymus supports the NeoThy immune-humanized mouse’s ability to produce adaptive immune responses.

### 3.5 NeoThy immune-humanized mice can produce antigen-specific and neutralizing anti-drug antibodies to therapeutic proteins

A primary endpoint in the development of new and biosimilar biological drugs is the ability to identify ADA responses to the drug, which could interfere with the assessment of pharmacokinetics and drug efficacy or result in drug safety issues. Our initial effort to develop these assays prioritized the assessment of nADAs, rather than bADAs, due to the limited volume of serum available from timepoints other than study termination. Specifically, it was difficult to obtain more than ~10 μL of serum at days 21 and 42, due to welfare limits on the total volume of blood that could be drawn. This—coupled with the use of serum for multiple assays (especially in saline-treated mice)—meant that we could not complete binding and neutralizing ADA assays for both drugs and that we could only evaluate ADAs at study beginning (day 0) and study end. The nADA assay for the monoclonal antibody product infliximab was relatively straightforward to develop; however, as IFN-β is not a monoclonal antibody, we found that it was not possible to conjugate a tag to the drug without disrupting the known ligand-binding site. Therefore, for IFN-β, we only developed a bADA assay.

**Figure 9:**
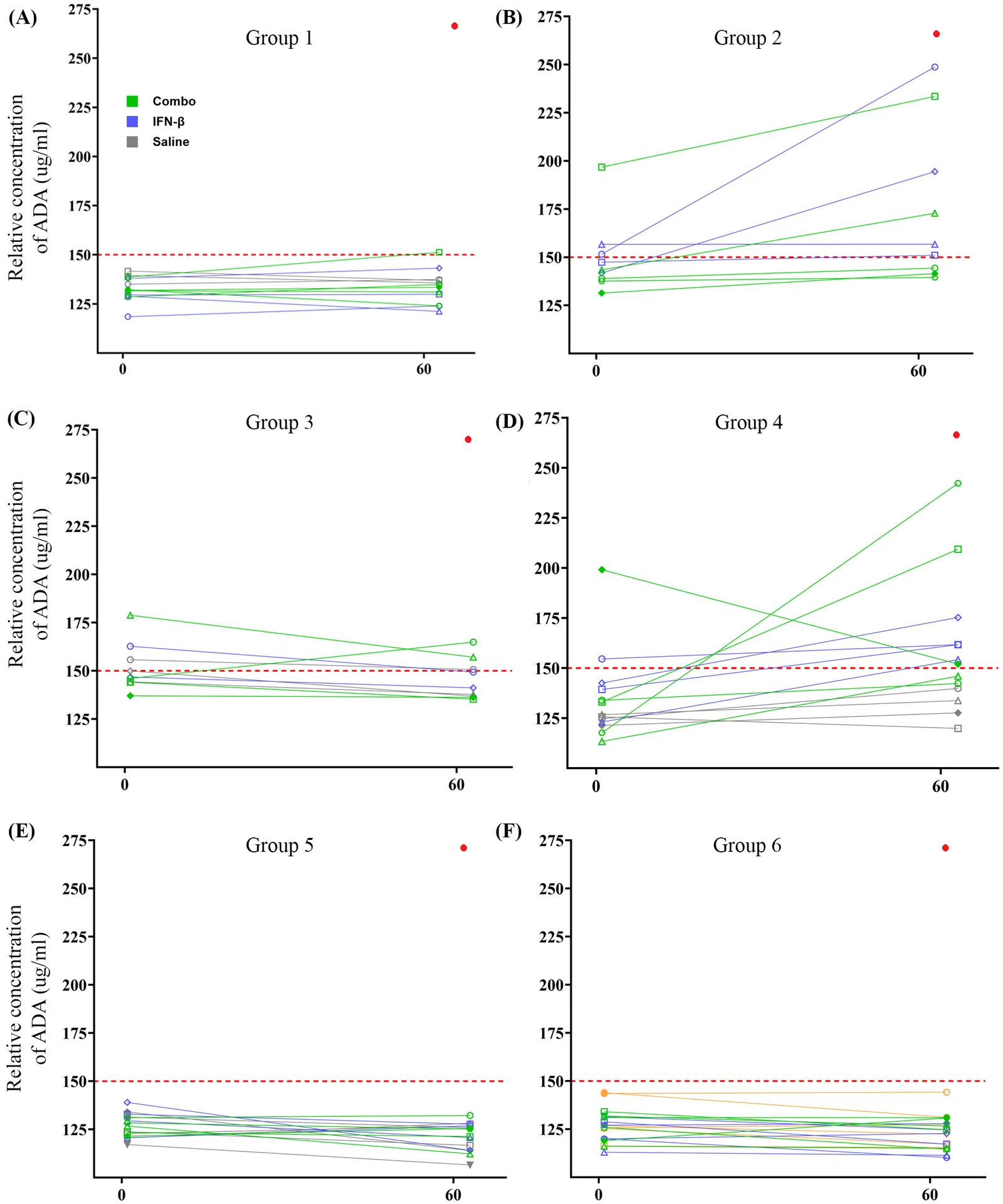
Measurement of anti-IFN-β IgG antibodies in serum. Binding ADA to IFN-β at day 0 and necropsy in humanized mice treated with IFN-β alone (purple, n = 25), combination therapy (green, IFN-β plus infliximab) (n = 29), or saline (gray, n = 22) from donors 1 – 6 (panels **A–F** respectively). The dashed red line indicates the assay cut point, above which samples are considered ADA-positive. The red dots represent a human serum positive control from a patient with confirmed antibodies against IFN-β. Some individual data lines overlap and may be visually indistinguishable. This is a semi-quantitative assay that utilizes a murine anti-human IFN-β standard curve to estimate relative values of human anti-IFN-β ADAs. ADA, anti-drug antibody.

**Figure 10:**
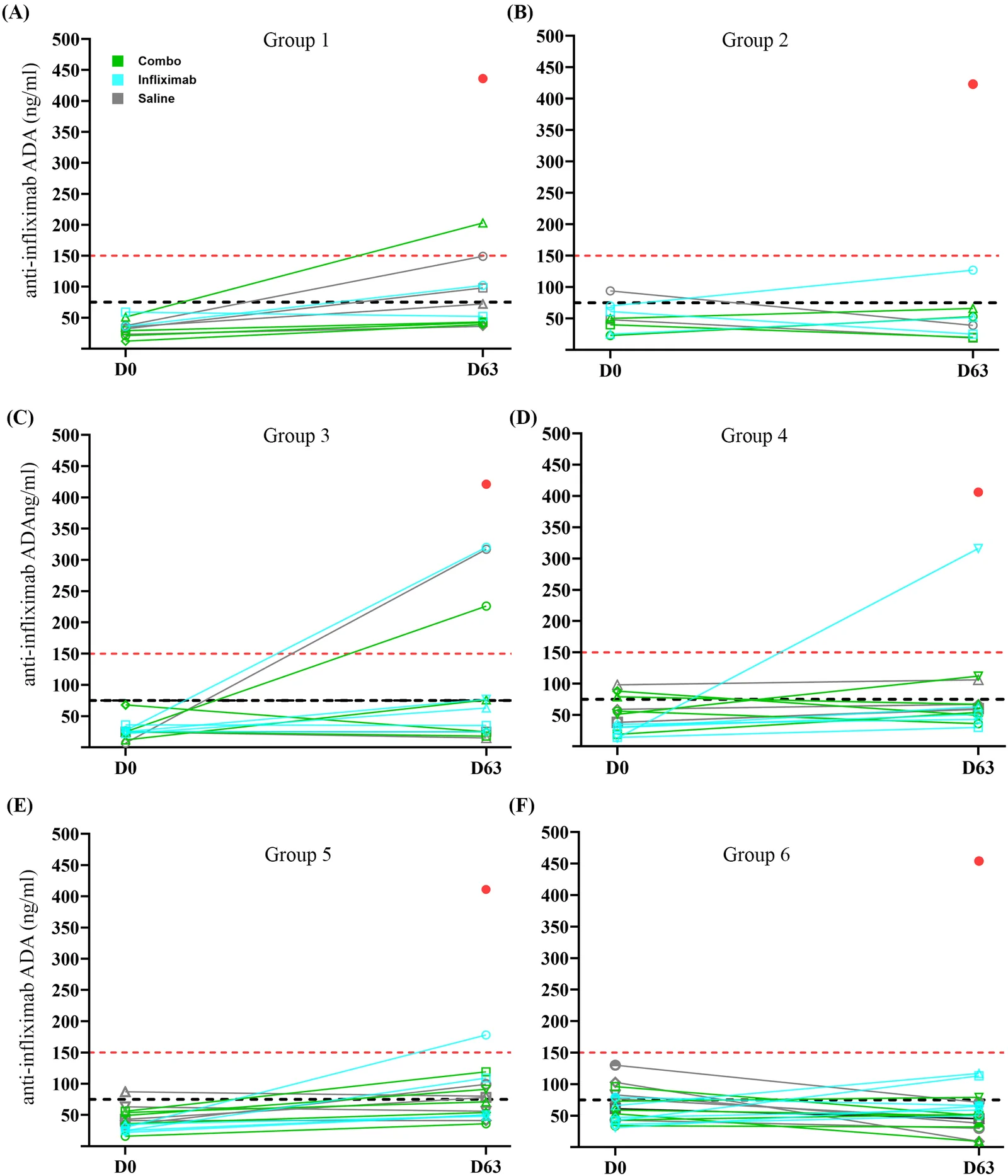
Measurement of neutralizing IgG antibodies against infliximab. Neutralizing ADA against infliximab at day 0 and necropsy in humanized mice treated with infliximab alone (light blue, n = 26), combination therapy (green, IFN-β plus infliximab) (n = 27), or saline (gray, n = 24) from donors 1 – 6 (panels **A–F** respectively). The dashed red line indicates the assay cut point, above which samples are considered ADA-positive, with the black dashed line showing the LLOQ. The red dots represent a human serum positive control from a patient with confirmed neutralizing antibodies to infliximab. Some individual data lines falling at or below the LLOQ may overlap and are visually indistinguishable. LLOQ, lower limit of quantitation.

To determine if the NeoThy immune-humanized mice that had been treated with IFN-β and/or infliximab at therapeutic levels could mount antigen-specific responses, we show results for the IFN-β ADA assay (**Figures 9A–F**) and the nADA assay against infliximab (**Figures 10A–F**). We found that multiple mice in groups 2 (**Figure 9B**) and 4 (**Figure 9D**), treated with either IFN-β alone or in combination with infliximab, developed antigen-specific ADAs. Mice in groups 1 (**Figure 9A**), 5 (**Figure 9E**), and 6 (**Figure 9F**) were produced with the same thymus, with group 1 produced at a different site but containing the identical HSC donors as groups 5 and 6, all of which consistently did not produce any ADAs to IFN-β. While several mice treated with IFN-β alone or in combination with infliximab in group 3 had ADA levels above the cut point (**Figure 9C**), levels were low, and they began above the cut point at day 0, suggesting that they were not treatment-induced. No mice treated with saline, in any group, produced ADAs to IFN-β.

As expected, fewer mice produced neutralizing ADAs to infliximab. We observed that mice treated with infliximab alone or in combination with IFN-β produced ADAs in groups 1, 3, 4, and 5. For reference, levels measured from human patients are shown (red dot on plots; **Figures 10C, D**), and while the levels in mice in these groups did not match those in human samples, they were sufficiently above the cut point to be readily measurable. One saline mouse in group 3 had measurable ADAs, which suggests that it produced this ADA in a non-specific manner.

We chose KLH as a positive control, as it is commonly used for this purpose in many immunological studies. It was expected that clear anti-KLH responses would be evident based on the increase in total IgM antibodies to 100–150 μg/mL at day 21 for many KLH-treated mice (**Figure 6**). However, we did not observe clear specific anti-KLH responses. Instead, we noted significant issues with non-specific antibody production by saline-treated mice against KLH for IgM (**Supplementary Figure 11**, left column), with minimal specific response in most KLH-treated mice. Even fewer responses were noted for anti-KLH IgG (**Supplementary Figure 11**, right column) antibody responses, with half of the positive mice also being saline-treated. It was also noted that almost every group with positive IgM responses had both KLH-treated and saline control mice demonstrating response, including at day 0, suggesting that broadly cross-reactive antibodies were being produced to this antigen and that it was not being recognized as foreign in the way that standard mouse strains would respond to it.

### 3.6 Positive ADA responses correlate with other assessments of immune activation

In support of the specificity of response to the drug products, proliferation experiments with LN cells showed specific responses to *ex vivo* stimulation from mice in group 4 (**Supplementary Figures 12A, C, E, G**), which were higher than those in control treatment and, in some cases, sixfold higher than even those in the positive control (ConA). In contrast, mice in group 6 that did not produce ADAs (**Supplementary Figures 12B, D, F, H**) showed minimal response to *ex vivo* stimulation other than to ConA.

To better understand the correlation between the production of ADAs in drug products and other measures of immune activation, we compared the responses across assays for all mice in groups 4 and 6 (**Tables 1A, B** respectively). All four mice treated with IFN-β produced bADAs to the drug, with three of five combo-treated mice developing bADAs and having *ex vivo* proliferative responses at or exceeding the assay cut point. Combo-treated mice had more mixed results, with some having concurrent proliferative responses matching their bADA results. Most ADA^+^ mice showed class switching and increased numbers of activated T cells in the LNs. Of note, mouse 2626, which showed an ADA^+^ result with positive proliferation, had almost no class switching or T-cell activation. This mouse is explained in **Figure 9D** as it was positive for anti-IFNβ ADAs at study start but decreased to the cut point over the course of the study, suggesting that this was a non-specific result, as it did not cause increases in T-cell activation or class switching. Mice in group 6 had no ADA^+^ results in any treated group, with a few mice showing proliferation results above the cut point. Most mice had positive T-cell proliferation responses to ConA that far exceeded the responses to any drug treatments, indicating the mice were capable of responding to stimulation. None of the mice in group 6 showed high amounts of class switching, and while activated T cells were present, their numbers were often much lower than those in group 4.

**Table 1A:** Comparison of responses in ADA-positive and ADA-negative mice for group 4.

Group 4			Proliferation^1^			IgG (ng/mL) @ necropsy			LN flow cytometry (absolute number)		
Mouse #	Tx	ADA	IFN-β	Inflix	ConA	IgG1	IgG2	IgG4	CD3+CD45RO+	CD3+HLA-DR+	CD3+CD38+
2626	Combo	Pos	6.21	<1.5×	<1.5×	1,167	3,417	8	434	7,990	391
2629	Combo	Neg	6.21	<1.5×	<1.5×	19,122	3,417	78	371,691	254,853	51,847
2662	Combo	Pos	<1.5×	<1.5×	<1.5×	324,494	315,209	111,727	157,306	167,664	19,633
2663	Combo	Neg	1.65	<1.5×	2.17	171,200	141,935	4,669	63,230	100,863	4,916
2670	Combo	Pos	<1.5×	<1.5×	<1.5×	462,903	1,249,200	1,085,600	69,689	217,374	13,967
2632	IFN-β	Pos	1.83	<1.5×	<1.5×	6,497	97,089	109	1,415,646	784,185	325,185
2646	IFN-β	Pos	1.50	<1.5×	1.5×	53,614	61,135	3,874	23,949	32,493	3,625
2668	IFN-β	Pos	1.58	<1.5×	1.77	14,849	3,417	1,165	336,487	264,817	168,547
2674*	IFN-β	Pos	1.60	<1.5×	2.63	173,870	52,008	73,073			
2630	Inflix	Neg	<1.5×	<1.5×	2.33	309,820	844,640	22,024	110,708	84,530	16,779
2636	Inflix	Neg	<1.5×	<1.5×	<1.5×	1,167	3,417	11	1,134	4,643	290
2637	Inflix	Neg	<1.5×	<1.5×	<1.5×	598,964	202,684	9,342	267,207	214,438	66,876
2671	Inflix	Neg	<1.5×	<1.5×	3.72	144,531	65,124	51,476	656,383	624,438	31,209
2673	Inflix	Pos	<1.5×	<1.5×	2.90	380,618	306,975	112,500	292,255	396,211	13,268
2627	Saline	Neg	<1.5×	<1.5×	<1.5×	113,853	101,019	4,646	5,983	12,866	820
2634	Saline	Neg	<1.5×	<1.5×	1.61	1,167	3,417	0	71,341	87,752	16,750
2645	Saline	Neg	<1.5×	<1.5×	<1.5×	46,785	4,660	531	134,153	130,035	18,240
2669	Saline	Neg	<1.5×	<1.5×	2.05	1,167	3,417	16	45,712	297,536	4,653

Comparison of immune responses in ADA-positive and ADA-negative mice from Group 4. ADA status; antigen-specific proliferation responses to IFN-β, infliximab, and ConA; IgG subclass concentrations at necropsy; and lymph node T-cell subset absolute counts were evaluated in individual Group 4 mice. Proliferation values represent fold increase over unstimulated conditions; values ≥1.50 were considered positive. Septic mice were excluded from flow cytometry analysis.

ADA, anti-drug antibody; LN, lymph node.

^1^ Fold increase over unstimulated; 1.50 increase or greater considered positive.

*Septic mouse; not included in flow cytometry analysis.

**Table 1B:** Comparison of responses in ADA-positive and ADA-negative mice for group 6.

Group 6			Proliferation^1^			IgG (ng/mL) at necropsy			LN flow cytometry (absolute number)		
Mouse #	Tx	ADA	IFN-β	Inflix	ConA	IgG1	IgG2	IgG4	CD3+CD45RO+	CD3+HLA-DR+	CD3+CD38+
2756	Combo	Neg	1.50	<1.5×	1.63	1,167	0	0	35,717	89,313	26,672
2767*	Combo	Neg	<1.5×	<1.5×	1.81	1,167	0	0			
2772	Combo	Neg	1.76	1.66	33.84	1,167	0	0	24,859	81,972	14,237
2776	Combo	Neg	<1.5×	<1.5×	<1.5×	1,167	0	0	39,058	9,939	40,760
2778	Combo	Neg	<1.5×	<1.5×	1.67	1,167	0	16	123,213	60,512	97,786
2783	Combo	Neg	<1.5×	<1.5×	2.79	1,167	0	13	161,961	69,873	162,729
2763	IFN-β	Neg	<1.5×	<1.5×	3.84	1,321	0	5	320,291	92,505	297,478
2764	IFN-β	Neg	1.69	<1.5×	3.26	684	2,241	194	66,860	203,648	21,906
2766	IFN-β	Neg	<1.5×	<1.5×	2.47	1,167	0	0	21,023	97,778	9,310
2770	IFN-β	Neg	<1.5×	<1.5×	<1.5×	1,167	0	0	49,643	77,080	37,896
2781	IFN-β	Neg	1.55	<1.5×	3.68	1,167	0	12	79,328	212,600	28,745
2782	IFN-β	Neg	<1.5×	<1.5×	<1.5×	1,167	0	11	135,547	118,992	77,301
2759	Inflix	Neg	<1.5×	<1.5×	1.61	256	0	5	36,919	61,235	18,905
2765	Inflix	Neg	<1.5×	<1.5×	3.53	1,167	0	0	63,352	188,831	17,139
2771	Inflix	Neg	<1.5×	<1.5×	2.64	1,167	0	0	24,978	62,683	9,295
2773	Inflix	Neg	<1.5×	<1.5×	3.01	1,167	0	0	30,668	127,815	4,948
2774	Inflix	Neg	<1.5×	<1.5×	<1.5×	1,167	2,848	226	7,143	31,421	2,003
2779	Inflix	Neg	<1.5×	<1.5×	<1.5×	1,167	0	13	20,499	21,436	23,904
2754	Saline	Neg	<1.5×	<1.5×	2.18	1,167	0	0	94,438	34,490	92,976
2755	Saline	Neg	<1.5×	<1.5×	<1.5×	1,167	0	0	51,122	30,291	53,662
2757	Saline	Neg	<1.5×	<1.5×	1.73	902	0	0	49,337	87,655	28,287
2758	Saline	Neg	<1.5×	<1.5×	<1.5×	1,167	0	0	27,705	83,003	14,824
2762	Saline	Neg	<1.5×	<1.5×	2.64	1,167	0	13	6,267	22,389	2,756
2769	Saline	Neg	<1.5×	<1.5×	<1.5×	1,167	0	0	5,206	19,326	1,981

Comparison of immune responses in ADA-positive and ADA-negative mice from Group 6. ADA status; antigen-specific proliferation responses to IFN-β, infliximab, and ConA; IgG subclass concentrations at necropsy; and lymph node T-cell subset absolute counts were evaluated in group 6 mice. Proliferation values represent fold increase over unstimulated conditions; values ≥1.50 were considered positive. Septic mice were excluded from flow cytometry analysis.

ADA, anti-drug antibody; LN, lymph node.

^1^ Fold increase over unstimulated; 1.50 increase or greater considered positive.

*Septic mouse; not included in flow cytometry analysis.

These data show that the ADA^+^ responses were specific to treatment and correlate with other assessments of immune activation.

## 4 Discussion

NeoThy™ immune-humanized mice were first reported in 2018 by Brown and colleagues (21). Their mice were produced using pediatric/neonatal thymus obtained from children undergoing heart surgery to repair congenital heart defects shortly after birth. Our laboratory’s interest in producing NeoThy immune-humanized mice began in 2018 following a restriction (30) on the use of fetal tissue to produce BLT immune-humanized mice. We then began work to use neonatal- or pediatric-derived thymus tissue in combination with either bone marrow- or cord blood-derived HSCs to produce these chimeric mice. To date, there has been no thorough interrogation of the NeoThy immune-humanized mouse’s ability to mount antigen-specific immune responses to biological drug products. In this proof-of-concept study, we sought to determine if they could produce adaptive immune responses to drug products known to induce immunogenicity in patients.

We show that NeoThy immune-humanized mice have full humanization of all tissues evaluated and that all major immune populations, including T cells, Tregs, B cells, monocytes, pDCs, mDCs, and NK cells, are present. Humanization levels (hCD45) were consistent among donors at the beginning of the study; however, two donor groups (4 and 5) showed increased numbers of human PBMCs at day 42, which impacted all treatment groups to which they had been randomized. Despite this increase, clear differences based on treatment could still be observed, indicating that donor variability can have an impact on results, just as would be found in human populations. This suggests that studies using immune-humanized mice should consider including a greater number of unique donors, for example, 6–10 individuals, to minimize the impact of a single individual.

It has been well-established that B-cell function in immune-humanized mice is deficient (17–20, 31). PBMC immune-humanized mice cannot mount antigen-specific responses, as they are short-lived and primarily have mature T cells and few B cells (16, 20, 31). CD34 HSC immune-humanized mice generally do have more naïve lymphocyte subsets and increased numbers of antigen-presenting cells; however, they have not shown the ability to produce antigen-specific responses to date (16, 20, 31). BLT immune-humanized mice have a fully engrafted immune system with a human thymus; however, only a few reports have demonstrated antigen-specific antibodies (8). Therefore, we wanted to determine if improved humoral immunity would be present in NeoThy immune-humanized mice, given the more mature human thymus and HSCs used to produce them, in combination with an immune-compromised mouse strain that expresses human IL-6. IL-6 is recognized as a proinflammatory cytokine, but it also plays an important role in class switching of B cells (32). Our data show clear evidence of class switching via flow cytometry and quantitation of human immunoglobulins by isotype. At necropsy, we found that the B cells in the lymph node were primarily CD27^+^IgD^+^ or CD27^+^IgD^−^, which would be expected if B cells were activated and class switching. The spleen and BM showed more abundant CD27^−^IgD^+^ naïve B cells, with the BM having a large population of CD27^+^IgD^+^ B cells. Interestingly, we found very few plasmablasts (B cell^+^/CD24^−^CD38hi^+^) in the LNs of saline-treated mice, but treated groups generally had plasmablasts present. We did not confirm the diversity of T-cell receptor and B-cell receptor repertoire as others have done (33); however, similar to their results, we show that CD27^+^IgD^−^ B cells are present, especially in the lymph node. This B-cell phenotype is known to signify class switching and maturation of B cells to an antigenic response (34).

Class switching was also confirmed using a human-specific multiplex assay for IgM and IgG_1–4_, such that total immunoglobulin isotype production was individually quantified. All mice, regardless of donor or treatment, produced measurable IgM, with very clear increases in total IgM at day 21 for most KLH-treated mice. Furthermore, IgG_1_ class switching was clearly observable for many infliximab- and combination-treated mice at day 21 in most donors, with the production of IgG_2–4_ that approached or exceeded the ULOQ in groups 2 and 4. Further support of proper humoral responses was observed with the histology of the lymph nodes showing clear presence of cortical lymphoid follicles. These data demonstrate that NeoThy immune-humanized mice clearly can produce class-switched antibody responses in measurable quantities.

Unlike other studies that used viruses or vaccines to demonstrate humoral immunity (33, 35), we chose to use drug products for infliximab and interferon-β, alone and in combination, to determine if antigen-specific antibodies could be produced. Previous studies have shown that infliximab, a chimeric monoclonal antibody targeting tumor necrosis factor-alpha, often induces ADA responses in autoimmune disease patients, ultimately impairing its therapeutic effectiveness (36–38). Similarly, specific IFN-β products, utilized in multiple sclerosis management, have been associated with the production of neutralizing antibodies that reduce their clinical utility (39). As clinical trials only evaluate the immunogenicity of individual biological drug products in patients, and many patients may now receive more than one during therapy, we chose to administer two immunogenic products together to determine if two immunogenic drugs would increase the incidence of ADAs to either or both of the products. NeoThy immune-humanized mice produced clear anti-IFN-β ADAs in mice from donor 2 and donor 4. Both IFN-β- and combination-treated mice had measurable antibody responses, with combination-treated mice having greater ADA levels than those treated with IFN-β alone in group 4, suggesting that combined dosing of immunogenic drugs could enhance ADA responses. Several mice in groups 3 and 4 produced neutralizing antibodies to infliximab, with levels that approached what was observed from a patient with neutralizing antibodies to the drug. We also show through a comparison of results from the different assays that the ADA responses observed in group 4 were consistent with the *ex vivo* proliferation, class switching, and immune activation of T cells. By comparison, group 6, which produced no ADAs, had strong responses to a mitogen in the *ex vivo* proliferation assay, but relatively weak class switching and limited immune activation in T cells. Taken together, NeoThy immune-humanized mice have the capacity to produce antigen-specific ADAs, and ADA results generally correlated with other measures of immune activation for these biological drug products.

The NeoThy immune-humanized mice used in this study were intentionally produced at both the FDA and Taconic to verify that similar responses would be observed if using the same starting material (HSCs and thymus). There were minor differences in the humanization protocols used by the two sites, and it is possible that they could contribute to differences in eventual humanization. The specific groups that were replicated were groups 1, 5, and 6. The thymus came from the same neonatal donor, with different HSC donors for groups 5 and 6, which are both found in group 1. While they consistently showed no evidence of ADAs for either drug, we observed two clear discrepancies. First, groups 5 and 6 both showed more clear increases in total IgM when treated with KLH as compared to group 1; second, group 1 produced increased levels of total IgG_1_ in both combination- and infliximab-treated mice at day 21, whereas groups 5 and 6 did not. It is unclear what the source of this difference is; however, all the mice were otherwise similar in their lack of specific ADA responses to infliximab and IFN-β. This demonstrates that while there were differences in some responses, the HSC donor showed the same ADA response, regardless of the difference in production site, affirming the reproducibility of the model. Other NeoThy immune-humanized mice produced by FDA (groups 2 and 3) and Taconic (group 4) included individuals that produced ADAs to the drug products, indicating that each location could successfully humanize mice that were capable of responding to the drug products and that the source of thymus/HSCs or production location was not a factor.

Another observation made in this study was that KLH does not appear to be a good positive control for chimeric mouse studies. KLH is often used as a positive control in studies measuring adaptive immune responses, and therefore, we expected to see robust responses to KLH in the antigen-specific IgM and IgG assays that we developed. We observed increased total IgM production at day 21 in most KLH-treated mice, with some showing positive responses in the anti-KLH IgM assay. However, in many groups, saline-treated mice were also positive in the anti-KLH IgM and IgG antibody assays, including at day 0. These saline-treated mice did not show cross-reactivity in the anti-infliximab or anti-IFN-β ADA assays. This suggests that immune-humanized mice may produce cross-reactive antibodies to KLH that are not developed in response to treatment. A precedent for this occurrence was recently shown for spontaneous IgE production in CD34 humanized mice, where class switching occurred in the absence of treatment (40). Lin et al. further showed that these spontaneously produced antibodies bound to commonly tested allergens such as seeds, cereals, and pollens, even though the mice were not exposed to them. These findings suggest that saline- or other control-treated mice from the same donor should always be tested for ADAs along with treated mice and that demonstration of increased total IgG isotypes and class switching is not sufficient to show specificity. It also suggests that due to the chimeric nature of the mice, more specific positive control compounds must be identified so that uniformly positive adaptive immune responses are observed in immune-humanized mouse studies going forward.

Our study had some logistical limitations. One limitation is that all the study mice were female. This was done for two reasons: first, group housing female mice is more straightforward than male mice, and second, females are generally more prone to immune-mediated disorders (41). The exclusion of males from the study means that differences in hormones and metabolism, which could impact results, were not addressed. Another issue is that some final treatment groups had very small sizes, with only two to four mice in a treatment group; this made it difficult to observe statistical differences between treatment groups due to the variability of individuals.

In this study, we attempted to address some of the variables associated with clear ADA production for human therapeutics. However, it was not possible to address many issues that relate to ADA prediction. Since our goal was to definitively show that ADAs could be produced to drug products at treatment-related doses and intervals, we chose products that have high rates of clinical immunogenicity (27, 28). Therefore, we had insufficient mice to add comparator drug products with low rates of immunogenicity to the study design to show no or low responses. Furthermore, while we tested human serum from patients who had discontinued these specific therapies, we only had two unique patient samples for each drug product, making broad comparisons in immunogenicity difficult. Overall responses observed within the same assay showed that some mice produced levels of ADAs in the same range as these patient samples, which is very promising but will require additional work to fully establish quantitative comparability.

The final limitation that should be addressed is that immune-humanized mice are chimeric animals, with a predominantly human immune system, but some innate murine components remain (16, 42). This can result in mice developing graft-versus-host disease (GvHD) or macrophage activation syndrome (MAS), based on the mouse strain used for humanization. Ensuring that responses observed directly relate to human immune responses and not an issue with the chimeric model itself is challenging. We addressed this by thoroughly evaluating histology for multiple tissues, determining if mice were septic at necropsy, and monitoring any other signs of model issues, such as murine tumors. GvHD would be evident in skin and intestinal tissue if present (43), and MAS generally leads to substantial infiltration of histiocytes and very low hematocrit values (44). We did not observe any mice with GvHD on histology, and very few mice had lesions consistent with MAS. Future immune-humanized mouse studies should include the evaluation of such baseline values to help determine if the responses observed are driven by the treatment article or are a result of the chimeric mouse.

In summary, this proof-of-concept study shows that NeoThy immune-humanized mice are fully humanized, with a broad repertoire of immune cells present. They are able to produce quantifiable amounts of all immunoglobulin isotypes and can produce anti-drug antibodies in quantities that are similar to those observed in patients. These data support further investigation of NeoThy immune-humanized mice as a model for the evaluation of immunogenicity. If further validated, they could have utility in applications such as biosimilar and originator drug product testing at various stages of drug development.

## 5 Conclusion

This proof-of-concept study demonstrates the promise of NeoThy immune-humanized mice in evaluating the immunogenicity of biological drugs. We have shown their ability to mount human-like immune responses, including the generation of ADAs and antigen-specific proliferation. To enhance their utility for preclinical immunogenicity assessment, further validation, refinement, and standardization are needed. These findings reinforce the role of humanized mouse models in immunogenicity research, potentially providing a platform for the assessment of ADA development and associated immune responses. By mimicking human immune dynamics, the NeoThy immune-humanized mouse model may provide a promising strategy for identifying and mitigating immunogenic risks in biological drug development.

## Data Availability

The datasets supporting the conclusions of this article are available in accordance with FDA policy and can be found at Open@fda.hhs.gov.
