# Supplementary material for: NeoThy™ immune-humanized mice can produce anti-drug antibodies to support immunogenicity assessment for biological drug products
Source: Front Immunol. 2026 Aug 12;17:1851898. doi: 10.3389/fimmu.2026.1851898 (PMC13509140; doi:10.3389/fimmu.2026.1851898)
Supplement: Supplementary file 1 [file DataSheet1.pdf]

Supplemental Table 1A. Thymus HLA type and donor information.

HLA type	Surgery Group 1	Surgery Group 2	Surgery Group 3	Surgery Group 4A	Surgery Group 4B	Surgery Group 5	Surgery Group 6
**Source**	Pediatric	Neonatal	Neonatal	Pediatric	Pediatric	Neonatal	Neonatal
**Donor ID**	A	B	C	D	E	A	A
**# matching alleles**	6-7	All	All	4	3	6	5
**Site of humanization**	FDA	FDA	FDA	Taconic	Taconic	Taconic	Taconic
**# mice made per surgery**	20	18	22	14	11	25	30
HLA-A	01:01	02:01	#	02	02	01:01	01:01
HLA-A	02:01	24:01	#	24	11	02:01	02:01
HLA-B	08:01	39:06	#	48	07	08:01	08:01
HLA-B	56:01	40:01	#	49	60	56:01	56:01
HLA-C	01:02	03:04	#	07	10	01:02	01:02
HLA-C	07:01	12:03	#	08	07	07:01	07:01
DQB1	02:02	05:03	#	-	-	02:02	02:02
DQB1	03:02	06:04	#	-	-	03:02	03:02
DPB1	04:01	03:01	#	-	-	04:01	04:01
DPB1	04:02	04:01	#	-	-	04:02	04:02
DRB1	03:01	13:02	#	04	04	03:01	03:01
DRB1	04:01	14:54	#	04	15	04:01	04:01
**Thymus donor information**	Same neonatal thymus tissue as Groups 5 and 6	Thymus and HSC originated from the same neonatal donor	Not HLA-typed; thymus and HSC originated from the same neonatal donor	Unique pediatric thymus donor paired with a unique HSC donor	Unique pediatric thymus donor paired with a unique HSC donor	Same neonatal thymus tissue as Groups 1 and 6	Same neonatal thymus tissue as Groups 1 and 5

HSC, hematopoietic stem cell.

“Neonatal” and “pediatric” are used to define source of tissue, not age of donor.

Dark blue font indicates HLA matching between the human thymus donor listed in Supplemental Table 1A and the $CD34^+$ HSC donor listed in Supplemental Table 1B. Pound (#) indicates HLA typing not available but matches HSC. Dash (-) indicates that HLA typing was not available or not reported for that locus/group.

Supplemental Table 1B. Hematopoietic stem cell HLA type and donor information.

HLA type	Surgery Group 1A	Surgery Group 1B	Surgery Group 2	Surgery Group 3	Surgery Group 4A	Surgery Group 4B	Surgery Group 5	Surgery Group 6
**Source**	Cord blood	Cord blood	Neonatal	Neonatal	Cord blood	Cord blood	Cord blood	Cord blood
**Donor ID**	F	G	B	C	H	I	F	G
**# matching alleles**	6	7	All	All	3	3	6	6
**Site of humanization**	FDA	FDA	FDA	FDA	Taconic	Taconic	Taconic	Taconic
**# mice made per HSC donor**	8*	12	18	22	14	11	25	30
HLA-A	01:01	01:01	02:01	#	02:01	02:01	01:01	01:01
HLA-A	02:01	68:01	24:01	#	26:01	23:01	02:01	68:01
HLA-B	08:01	08:01	39:06	#	27:05	51:02	08:01	08:01
HLA-B	15:01	15:18	40:01	#	44:02	58:01	15:01	15:18
HLA-C	03:01	07:01	03:04	#	02:02	07:01	03:01	07:01
HLA-C	07:01	07:04	12:03	#	07:04	08:01	07:01	07:04
DQB1	02:01	02:01	05:03	#	05:01	02:01	02:01	02:01
DQB1	06:02	06:02	06:04	#	06:03	03:02	06:02	06:02
DPB1	04:01	04:01	03:01	#	04:01	04:01	04:01	04:01
DPB1	10:01	04:01	04:01	#	04:02	04:02	10:01	04:01
DRB1	03:01	15:01	13:02	#	04:08	03:01	03:01	15:01
DRB1	15:01	03:01	14:54	#	13:02	04:07	15:01	03:01
**HSC donor information**	Shared with Group 5	Shared with Group 6	Thymus and HSC originated from the same neonatal donor	Not HLA-typed; thymus and HSC originated from the same neonatal donor	Unique HSC donor paired with a different pediatric thymus donor	Unique HSC donor paired with a different pediatric thymus donor	Same HSC donor as Group 1 HSC donor 1	Same HSC donor as Group 1 HSC donor 2

HSC, hematopoietic stem cell.

***** One mouse in this group was produced with HSC from thymus donor A (100% match) and is not shown separately.

Dark blue font indicates HLA matching between the human thymus donor listed in Supplemental Table 1A and the CD34$^+$ HSC donor listed in Supplemental Table 1B. Pound (#) indicates HLA typing not available but matches HSC. Dash (-) indicates that HLA typing was not available or not reported for that locus/group.
