# Supplementary material for: NeoThy™ immune-humanized mice can produce anti-drug antibodies to support immunogenicity assessment for biological drug products
Source: Front Immunol. 2026 Aug 12;17:1851898. doi: 10.3389/fimmu.2026.1851898 (PMC13509140; doi:10.3389/fimmu.2026.1851898)
Supplement: Supplementary file 2 [file DataSheet2.pdf]

Supplemental Table 2. Antibodies used for lineage flow cytometry.

**Antibody**	**Clone**	**Species**	**Vendor**
CD3	HIT3a	human	Biolegend
CD4	RPA-T4	human	Biolegend
CD8a	HIT8a	human	Invitrogen
CD11c	SHCL-3	human	BD Biosciences
CD11c	B-ly6	human	BD Biosciences
CD14	HCD14	human	Biolegend
CD16	3G8	human	BD Biosciences
CD16	eBioCB16	human	Invitrogen
CD19	HIB19	human	Biolegend
CD20	2H7	human	Biolegend
CD24	ML5	human	BD Biosciences
CD25	BC96	human	Biolegend
CD27	O323	human	Biolegend
CD38	HIT2	human	BD Biosciences
CD40	5C3	human	Biolegend
CD45	HI30	human	Biolegend
CD45	30-F11	murine	Biolegend; Invitrogen
CD45RA	HI100	human	Invitrogen
CD45RO	UCHL1	human	BD Biosciences
CD56	NCAM16.2	human	BD Biosciences
CD56	HCD56	human	Biolegend
CD66b	G10F5	human	Biolegend
CD80	2D10	human	Biolegend
CD80	2D10.4	human	Invitrogen
CD123	6H6	human	Biolegend
CD127	A019D5	human	Biolegend
CD154	24-31	human	Biolegend; Invitrogen
CD197	3D12	human	BD Biosciences; Invitrogen
HLA-DR	L243	human	Biolegend
IgD	IA6-2	human	Biolegend
