# Supplementary material for: NeoThy™ immune-humanized mice can produce anti-drug antibodies to support immunogenicity assessment for biological drug products
Source: Front Immunol. 2026 Aug 12;17:1851898. doi: 10.3389/fimmu.2026.1851898 (PMC13509140; doi:10.3389/fimmu.2026.1851898)
Supplement: Supplementary file 3 [file DataSheet3.pdf]

Supplemental Table 3. Individual mouse information by surgery group.

Mouse number	Surgery Group	Sex	Age at study start (weeks)	Treatment	Study day euthanized	Thymus donor	HSC donor	Comment (if excluded from flow data analysis)
**2599**	1	F	35	Infliximab	Day 63	A	G	
**2607**	1	F	35	Infliximab	Day 63	A	F	
**2613**	1	F	35	Infliximab	Day 63	A	G	Septic
**2617**	1	F	35	Infliximab	Day 63	A	F	Septic
**2602**	1	F	35	IFNβ	Day 63	A	G	
**2603**	1	F	35	IFNβ	Day 63	A	G	
**2611**	1	F	35	IFNβ	Day 63	A	G	
**2619**	1	F	35	IFNβ	Day 63	A	F	
**2605**	1	F	35	Combo	Day 63	A	F	
**2608**	1	F	35	Combo	Day 63	A	A	
**2610**	1	F	35	Combo	Day 63	A	G	
**2614**	1	F	35	Combo	Day 63	A	G	
**2604**	1	F	35	KLH	Day 28	A	G	Murine leukemia
**2606**	1	F	35	KLH	Day 63	A	F	
**2612**	1	F	35	KLH	Day 63	A	G	
**2616**	1	F	35	KLH	Day 63	A	F	
**2600**	1	F	35	Saline	Day 63	A	G	
**2615**	1	F	35	Saline	Day 63	A	G	
**2621**	1	F	35	Saline	Day 63	A	F	
**2598**	1	F	35	Saline	Day 63	A	G	
	**n=20**							
**2578**	2	F	34	Infliximab	Day 63	B	B	
**2582**	2	F	34	Infliximab	Day 63	B	B	
**2587**	2	F	34	Infliximab	Day 21	B	B	
**2591**	2	F	34	Infliximab	Day 56	B	B	
**2573**	2	F	34	IFNβ	Day 63	B	B	
**2576**	2	F	34	IFNβ	Day 63	B	B	
**2583**	2	F	34	IFNβ	Day 49	B	B	
**2586**	2	F	34	IFNβ	Day 63	B	B	
**2572**	2	F	34	Combo	Day 63	B	B	
**2577**	2	F	34	Combo	Day 63	B	B	Septic
**2584**	2	F	34	Combo	Day 63	B	B	
**2588**	2	F	34	Combo	Day 33	B	B	
**2581**	2	F	34	KLH	Day 63	B	B	
**2590**	2	F	34	KLH	Day 43	B	B	
**2592**	2	F	34	KLH	Day 63	B	B	
**2579**	2	F	34	Saline	Day 63	B	B	
**2596**	2	F	34	Saline	Day 33	B	B	
**2594**	2	F	34	Saline	Day 63	B	B	
	**n=18**							
**2539**	3	F	34	Combo	Day 29	C	C	
**2550**	3	F	34	Combo	Day 63	C	C	
**2553**	3	F	34	Combo	Day 63	C	C	
**2554**	3	F	34	Combo	Day 63	C	C	Septic
**2556**	3	F	34	Combo	Day 19	C	C	
**2544**	3	F	34	Infliximab	Day 63	C	C	
**2547**	3	F	34	Infliximab	Day 63	C	C	
**2551**	3	F	34	Infliximab	Day 63	C	C	
**2557**	3	F	34	Infliximab	Day 63	C	C	Septic
**2559**	3	F	34	Infliximab	Day 63	C	C	
**2546**	3	F	34	IFNβ	Day 23	C	C	
**2548**	3	F	34	IFNβ	Day 63	C	C	
**2549**	3	F	34	IFNβ	Day 63	C	C	
**2558**	3	F	34	IFNβ	Day 23	C	C	
**2536**	3	F	34	KLH	Day 63	C	C	
**2541**	3	F	34	KLH	Day 63	C	C	
**2543**	3	F	34	KLH	Day 63	C	C	
**2555**	3	F	34	KLH	Day 63	C	C	
**2537**	3	F	34	Saline	Day 27	C	C	
**2542**	3	F	34	Saline	Day 34	C	C	
**2552**	3	F	34	Saline	Day 63	C	C	
**2560**	3	F	34	Saline	Day 63	C	C	
	**n=22**							

Mouse number	Surgery Group	Sex	Age at study start (weeks)	Treatment	Study day euthanized	Thymus donor	HSC donor	Comment (if excluded from flow data analysis)
**2626**	4	F	20	Combo	Day 59	D	H	
**2629**	4	F	20	Combo	Day 59	D	H	
**2662**	4	F	20	Combo	Day 63	E	I	
**2663**	4	F	20	Combo	Day 63	E	I	
**2670**	4	F	20	Combo	Day 63	E	I	
**2630**	4	F	20	Infliximab	Day 59	D	H	
**2636**	4	F	20	Infliximab	Day 59	D	H	
**2637**	4	F	20	Infliximab	Day 63	D	H	
**2671**	4	F	20	Infliximab	Day 63	E	I	
**2673**	4	F	20	Infliximab	Day 63	E	I	
**2632**	4	F	20	IFNβ	Day 59	D	H	
**2633**	4	F	20	IFNβ	Day 54	D	H	
**2646**	4	F	20	IFNβ	Day 59	D	H	
**2668**	4	F	20	IFNβ	Day 63	E	I	
**2674**	4	F	20	IFNβ	Day 63	E	I	Septic
**2631**	4	F	20	KLH	Day 59	D	H	
**2639**	4	F	20	KLH	Day 59	D	H	
**2644**	4	F	20	KLH	Day 59	D	H	
**2659**	4	F	20	KLH	Day 63	E	I	
**2677**	4	F	20	KLH	Day 63	E	I	
**2627**	4	F	20	Saline	Day 59	D	H	
**2634**	4	F	20	Saline	Day 59	D	H	
**2645**	4	F	20	Saline	Day 63	D	H	
**2665**	4	F	20	Saline	Day 28	E	I	
**2669**	4	F	20	Saline	Day 63	E	I	
	**n=25**							
**2747**	5	F	22	Combo	Day 59	A	F	Septic
**2725**	5	F	22	Combo	Day 59	A	F	
**2731**	5	F	22	Combo	Day 59	A	F	
**2742**	5	F	22	Combo	Day 63	A	F	
**2733**	5	F	22	Combo	Day 63	A	F	
**2752**	5	F	22	Infliximab	Day 59	A	F	
**2748**	5	F	22	Infliximab	Day 59	A	F	Septic
**2737**	5	F	22	Infliximab	Day 59	A	F	
**2732**	5	F	22	Infliximab	Day 63	A	F	
**2734**	5	F	22	Infliximab	Day 63	A	F	
**2751**	5	F	22	IFNβ	Day 59	A	F	
**2730**	5	F	22	IFNβ	Day 59	A	F	
**2726**	5	F	22	IFNβ	Day 63	A	F	
**2729**	5	F	22	IFNβ	Day 63	A	F	
**2738**	5	F	22	IFNβ	Day 63	A	F	
**2735**	5	F	22	KLH	Day 59	A	F	
**2753**	5	F	22	KLH	Day 59	A	F	
**2741**	5	F	22	KLH	Day 59	A	F	
**2740**	5	F	22	KLH	Day 63	A	F	
**2743**	5	F	22	KLH	Day 63	A	F	
**2746**	5	F	22	Saline	Day 59	A	F	Septic
**2750**	5	F	22	Saline	Day 59	A	F	Septic
**2749**	5	F	22	Saline	Day 63	A	F	
**2739**	5	F	22	Saline	Day 63	A	F	
**2744**	5	F	22	Saline	Day 63	A	F	
	**n=25**							
**2756**	6	F	22	Combo	Day 63	A	G	
**2767**	6	F	22	Combo	Day 63	A	G	Septic
**2772**	6	F	22	Combo	Day 63	A	G	
**2776**	6	F	22	Combo	Day 63	A	G	
**2778**	6	F	22	Combo	Day 63	A	G	
**2783**	6	F	22	Combo	Day 63	A	G	
**2759**	6	F	22	Infliximab	Day 63	A	G	
**2765**	6	F	22	Infliximab	Day 63	A	G	
**2771**	6	F	22	Infliximab	Day 63	A	G	
**2773**	6	F	22	Infliximab	Day 63	A	G	
**2774**	6	F	22	Infliximab	Day 63	A	G	
**2779**	6	F	22	Infliximab	Day 63	A	G	
**2763**	6	F	22	IFNβ	Day 63	A	G	

Mouse number	Surgery Group	Sex	Age at study start (weeks)	Treatment	Study day euthanized	Thymus donor	HSC donor	Comment (if excluded from flow data analysis)
**2764**	6	F	22	IFNβ	Day 63	A	G	
**2766**	6	F	22	IFNβ	Day 63	A	G	
**2770**	6	F	22	IFNβ	Day 63	A	G	
**2781**	6	F	22	IFNβ	Day 63	A	G	
**2782**	6	F	22	IFNβ	Day 63	A	G	
**2760**	6	F	22	KLH	Day 63	A	G	
**2761**	6	F	22	KLH	Day 63	A	G	
**2768**	6	F	22	KLH	Day 63	A	G	Septic
**2775**	6	F	22	KLH	Day 63	A	G	
**2777**	6	F	22	KLH	Day 63	A	G	Septic
**2780**	6	F	22	KLH	Day 63	A	G	
**2754**	6	F	22	Saline	Day 63	A	G	
**2755**	6	F	22	Saline	Day 63	A	G	
**2757**	6	F	22	Saline	Day 63	A	G	
**2758**	6	F	22	Saline	Day 63	A	G	
**2762**	6	F	22	Saline	Day 63	A	G	
**2769**	6	F	22	Saline	Day 63	A	G	
	**n=30**							

Footnotes:
Groups 1, 5, and 6 used the same neonatal thymus tissue.
Group 1 used the same HSC donor set as Groups 5 and 6.
Group 3 was not HLA-typed; thymus and HSC originated from the same neonatal donor.
Group 4 used two unique HSC donors, each with a different pediatric thymus donor.
Full HLA profiles are reported separately in Supplemental Table 1A (thymus HLA type) and Supplemental Table 1B (HSC HLA type).
