# Supplementary material for: NeoThy™ immune-humanized mice can produce anti-drug antibodies to support immunogenicity assessment for biological drug products
Source: Front Immunol. 2026 Aug 12;17:1851898. doi: 10.3389/fimmu.2026.1851898 (PMC13509140; doi:10.3389/fimmu.2026.1851898)
Supplement: Supplementary file 4 [file DataSheet4.pdf]

Supplemental Figure 4. Flow gating definitions.

Category	Cell population / parameter	Surface marker definition
Leukocytes	Human leukocytes	hCD45+ mCD45−
T cells	CD3+ T cells	hCD45+ CD3+
T cells	CD4+ T cells	hCD45+ CD3+ CD4+
T cells	T-regulatory cells (T-reg)	hCD45+ CD3+ CD4+ CD25+ CD127−
T cells	CD8+ T cells	hCD45+ CD3+ CD8a+ CD4-
T cells	CD4+CD8+ T cells	hCD45+ CD3+ CD4+ CD8a+
T cells	CD3+CD45RA+ T cells	hCD45+ CD3+ CD45RA+
T cells	CD3+CD45RO+ T cells	hCD45+ CD3+ CD45RO+
T cells	Activated CD3+HLA-DR+ T cells	hCD45+ CD3+ HLA-DR+CD38-
T cells	Activated CD3+CD38+ T cells	hCD45+ CD3+ CD38+HLA-DR-
T cells	CD3+CD80+ T cells	hCD45+ CD3+ CD80+
T cells	CD3+CD25+ T cells	hCD45+ CD3+ CD25+
T cells	CD3+CD154+ T cells	hCD45+ CD3+ CD154+
Myeloid cells	Myeloid HLA-DR+ cells	hCD45+ CD3- CD19/CD20- CD14- CD16+ HLA-DR+
Dendritic cells	Myeloid dendritic cells (mDC)	hCD45+ CD3- CD19/CD20- CD14- CD16+ HLA-DR+ CD11c+ CD123−
Dendritic cells	Plasmacytoid dendritic cells (pDC)	hCD45+ CD3- CD19/CD20- CD14- CD16+ HLA-DR+ CD123+ CD11c−
Granulocytes	Basophils	hCD45+ CD3- CD19/CD20- HLA-DR− CD11c+
Granulocytes	Eosinophils	hCD45+ CD3- CD19/CD20- CD66b+ HLA-DR− CD16−/low
Granulocytes	Neutrophils	hCD45+ CD3- CD19/CD20- CD66b+ CD16+ HLA-DR−
Monocytes	Classical monocytes	hCD45+ CD3-CD19/CD20- CD14+ CD16−
Monocytes	CD14+CD16+ monocytes	hCD45+ CD3-CD19/CD20- CD14+ CD16+
Monocytes	Total monocytes	hCD45+ CD3- CD19/CD20- CD14+ CD16- CD14+ CD16+
Monocytes	HLA-DR+ monocytes	hCD45+ CD3-CD19/CD20- CD14+ CD16− HLA-DR+
Monocytes	CD80+ monocytes	hCD45+ CD3-CD19/CD20- CD14+ CD16− CD80+
B cells	Total B cells	hCD45+ CD3- CD19/CD20+
B cells	CD45RA+ B cells	hCD45+ CD3- CD19/CD20+ CD45RA+
B cells	CD45RO+ B cells	hCD45+ CD3- CD19+ CD20+ CD45RO+
B cells	Class-switched memory B cells	hCD45+ CD3- CD19/CD20+ CD27+ IgD−
B cells	Non-switched memory B cells	hCD45+ CD3- CD19/CD20+ CD27+ IgD+
B cells	Naive B cells	hCD45+ CD3- CD19/CD20+ CD27− IgD+
B cells	Double-negative B cells	hCD45+ CD3- CD19/CD20+ CD27− IgD−
B cells	CD24+CD38− B cells	hCD45+ CD3- CD19/CD20+ CD24+ CD38−
B cells	Plasmablasts	hCD45+ CD3- CD19/CD20+ CD24+ CD38+high
B cells	CD24+CD38+ B cells	hCD45+ CD3- CD19/CD20+ CD24+ CD38+
B cells	CD24−CD38+ B cells	hCD45+ CD3- CD19/CD20+ CD24− CD38+low
B cells	HLA-DR+ B cells	hCD45+ CD3- CD19/CD20+ HLA-DR+
B cells	CD80+ B cells	hCD45+ CD3- CD19/CD20+ CD80+
NK/NKT cells	NKT cells	hCD45+ CD3+ CD56+
NK/NKT cells	Total NK cells	hCD45+ CD3− CD56+
NK/NKT cells	CD56+CD16+ NK cells	hCD45+ CD3− CD56+ CD16+
NK/NKT cells	CD56+CD16− NK cells	hCD45+ CD3− CD56+ CD16−
